# Anxiolytic and Memory Protective Effects of Withanolide D Isolated From *Acnistus arborescens* in Adult Zebrafish

**DOI:** 10.1002/cbdv.202502272

**Published:** 2026-04-30

**Authors:** Cléia Rocha de Sousa Feitosa, Joilna Alves Da Silva, Jéssica Bezerra Maciel, Ivana Carneiro Romão, Thais Rocha Cavalcante, Francisco Anderson Nascimento Barros, Nicole de Abreu Bandeira, Emmanuel Silva Marinho, Jane Eire Silva Alencar De Menezes, Maria Kueirislene Amâncio Ferreira, Antonio Wlisses Da Silva, Márcia Machado Marinho, Matheus Nunes Da Rocha, Otília Deusdênia Loiola Pessoa, Pedro Henrique Jatai Batista, Andreia Ferreira De Castro Gomes, Alexandre Magno Rodrigues Teixeira, Hélcio Silva Dos Santos

**Affiliations:** ^1^ Graduate Program in Biological Chemistry Universidade Regional do Cariri Crato Brazil; ^2^ Graduate Program in Natural Sciences Campus Do Itaperi State University of Ceará Fortaleza Brazil; ^3^ Chemistry Course Science and Technology Center Vale Do Acaraú State University Sobral Brazil; ^4^ Chemistry Course Science and Technology Center State University of Ceará Fortaleza Brazil; ^5^ Graduate Program in Chemistry Universidade Federal do Ceará Fortaleza Brazil; ^6^ Centre of Molecular and Environmental Biology (CBMA)/Aquatic Research Network (ARNET) Associate Laboratory Campus De Gualtar University of Minho Braga Portugal

**Keywords:** *Acnistus arborescens*, anxiety, *Danio rerio*, learning, withanolide D

## Abstract

This study evaluated the anxiolytic and memory‐protective effects of withanolide D in adult zebrafish (Danio rerio). The compound was administered at doses of 4, 20, and 40 mg/kg and showed no signs of toxicity after 96 h of observation at any of the doses tested. In the open field test, withanolide D reduced locomotor activity, indicating a sedative‐like effect. Nevertheless, in the light/dark test, doses of 20 and 40 mg/kg produced an anxiolytic‐like response, suggesting that anxiolytic and motor effects may partially overlap depending on dose. The anxiolytic effect observed at the minimum effective dose (20 mg/kg) was reversed by flumazenil (FMZ), supporting the involvement of a benzodiazepine‐sensitive GABA_A_ modulatory domain. Furthermore, withanolide D, at a dose of 4 mg/kg, prevented ethanol‐induced memory impairment in the inhibitory avoidance task, suggesting a protective effect on memory consolidation. Molecular docking analyses revealed favorable interactions of withanolide D at the extracellular α1γ2 interface of the GABA_A_ receptor, supporting a putative allosteric interaction in a functionally related modulatory region rather than definitive occupation of the classical diazepam site. Consistently, normal mode analysis (NMA) showed that withanolide D increases receptor mobility compared to diazepam, with RMSF values reaching up to 0.98 Å, indicating enhanced structural flexibility and dynamic modulation of the protein. Thus, these findings suggest that withanolide D has therapeutic potential for the treatment of anxiety, in addition to providing protective effects on memory.

## Graphical abstract

Withanolide D Exhibits Anxiolytic and Memory‐Protective Effects.

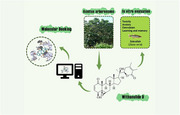

## 1 Introduction

Natural products remain a central source for drug discovery, particularly due to their structural diversity and ability to modulate multiple biological targets with potentially lower toxicity and cost compared to synthetic compounds [1, 2]. Among these, medicinal plants have been extensively investigated as reservoirs of bioactive secondary metabolites, many of which have already led to effective therapeutic agents for the treatment of human diseases [3, 4].

Within this context, plants of the family Solanaceae are recognized for producing a wide range of steroidal metabolites, notably withanolides, a class of naturally occurring polyoxygenated C28 steroidal lactones characterized by an ergostane skeleton and structural variability in oxygenation patterns and degrees of unsaturation [6]. Approximately 90% of known withanolides possess a ketone group at C‐1, a structural feature associated with relevant biological activities [6].

Withanolides have attracted increasing scientific interest due to their broad spectrum of pharmacological effects, including anti‐inflammatory, antitumor, immunomodulatory, neuroprotective, and cholinesterase inhibitory activities [4, 7, 8, 9, 10, 11]. In the context of central nervous system (CNS) disorders, these compounds have demonstrated anxiolytic‐like, neuroprotective, and cognition‐modulating effects in both in vitro and in vivo studies, which have motivated further investigations using experimental and computational approaches [9, 10, 11, 12, 13, 14, 15].

Despite these promising effects, it is important to note that withanolides are not devoid of limitations, particularly regarding dose‐dependent toxicity, which has been reported for some members of this class, especially those isolated from *Withania somnifera* [12, 16, 17, 18, 19]. Such findings reinforce the need to investigate alternative botanical sources of withanolides, aiming to identify structurally distinct molecules with improved safety and pharmacological profiles.

In this regard, *Acnistus arborescens*, a Solanaceae species native to northeastern Brazil [5], represents a less explored but promising source of withanolides. Although most pharmacological studies on withanolides focus on *W. somnifera*, emerging evidence suggests that withanolides isolated from other Solanaceae species may exhibit distinct biological activities and toxicity profiles due to subtle structural differences [4, 6, 7, 8, 9, 10, 11]. However, detailed phytochemical characterization and in vivo behavioral evaluations of A. arborescens remain scarce, particularly concerning CNS‐related effects.

Anxiety disorders affect millions of individuals worldwide and are commonly treated with benzodiazepines and selective serotonin reuptake inhibitors (SSRIs) [21]. Although effective, these drugs are associated with significant adverse effects, including sedation, tolerance, dependence, cognitive impairment, and withdrawal symptoms following prolonged use [22, 23, 24]. Similarly, neurodegenerative diseases such as Alzheimer's disease, characterized by progressive cognitive decline and memory loss, represent a major global health burden [25]. Current therapeutic strategies for Alzheimer's disease include cholinesterase enzyme inhibitors (ChEIs); however, their limited efficacy and side effects highlight the need for new compounds with multitarget activity and improved safety [10, 26, 27].

To investigate novel neuroactive agents with anxiolytic and cognitive‐modulating properties, validated animal models are essential. While rodents are traditionally employed, their high maintenance costs and ethical constraints have encouraged the use of alternative models such as the zebrafish (*Danio rerio*) [21, 28, 29]. Adult zebrafish present well‐characterized behavioral paradigms relevant to anxiety and memory, substantial neurochemical and genetic homology with humans (approximately 70% for neurotransmitter systems), and practical advantages including low cost, rapid drug absorption by immersion, and compliance with the 3Rs principles [30, 31].

Therefore, the present study aimed to investigate the anxiolytic‐like effects of withanolide D isolated from A. arborescens, elucidate its possible mechanism of action, and evaluate its impact on memory performance using adult zebrafish as an experimental model. By exploring a non‐conventional botanical source of withanolides, this work seeks to contribute to the identification of neuroactive compounds with therapeutic potential and improved safety profiles for CNS disorders.

## 2 Results and Discussion

Although there are many studies analyzing the pharmacological potential of other withanolides isolated from plants, mainly related to cancer, anti‐inflammatory, anti‐stress, immunomodulatory, neuroprotective, among others [4, 6], this is believed to be the first to investigate the anxiolytic activity and memory protection of withanolide D, a withanolide isolated from *A. arborescens* in an experimental model of adult zebrafish.

### 2.1 Characterization

**FIGURE 1:**
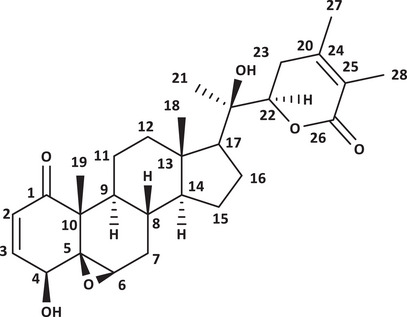
Structural representation of withanolide D isolated from *A. arborescens*.

Withanolide D (Figure 1) was isolated from the acetone extract of *A. arborescens* leaves. Its high‐resolution mass spectrum (Figure S1) showed a peak at m/z 493.2595 [M]^+^ compatible with the formula C_28_H_38_O_6_, which showed IDH = 10. The ^13^C NMR spectrum (Figure S2) of the chemical shifts of withanolide D showed the presence of eight non‐hydrogenated carbons, including two carbonyls at *δ*_C_ 202.9 (C‐1) and 166.8 (C‐26), the latter being characteristic of conjugated *δ*‐lactone, very common in withanolides. Signals referring to nine methine carbons were also observed, including four oxygenated carbons: *δ*_C_ 60.4 (C‐6), 70.7 (C‐4), 145.3 (C‐3), as well as six methylene carbons at *δ*_C_ 60.4 (C‐7), 21.4 (C‐11), 40.1 (C‐12), 24.5 (C‐15), 21.9 (C‐16), and 32.0 (C‐23) and five methyl carbons at *δ*_C_ 14.2 (C‐18), 20.5 (C‐19), 22.8 (C‐21), 12.9 (C‐27), and 17.4 (C‐28). In the ^1^H NMR spectrum (Figure S3) of withanolide D, signals corresponding to two olefinic hydrogens were observed at *δ*_H_ 6.47 (d;1H *J* = 9.8 Hz) and 7.25 (dd;1H *J* = 6.3; 9.8 Hz), referring to olefinic hydrogens 2 and 3, respectively, as well as a doublet at *δ*_H_ 4.0 (d; 1H *J* = 5.2 Hz) referring to hydrogen 4, a broad singlet at *δ*_H_ 3.26 (s 1H) for hydrogen 6 and 4.40 (dd; 1H *J* = 3.1; 8.6 Hz) for hydrogen 22, corresponding to hydrogens bound to oxygenated carbons. Other simplexes were observed corresponding to methyl groups in *δ*H 1.84 (3H‐21), 1.41 (3H‐19), 1.85 (3H‐28), 1.94 (3H‐27), and 1.10 (3H‐18), bound to carbon in *δ*C 22.8 (C‐21), 20.5 (C‐19), 17.4 (C‐28), 12.9 (C‐27), and 14.2 (C‐18), respectively. The NMR values of isolated withanolide D were in good agreement with reported values [12, 16].

### 2.2 Acute Toxicity 96 h

Withanolide D administered intraperitoneally to groups of adult zebrafish at doses of 4, 20, and 40 mg/kg (*n* = 6/group; 20 µL; i.p.) showed no evidence of toxicity (Table 1). No mortality was observed during the 96 h monitoring period at any of the doses tested (LD50>40 mg/kg, Table 1), and no apparent anatomical changes were detected throughout this interval (*p* > 0.05). These findings corroborate the preclinical safety of withanolides, which represents a fundamental step in the search for new drug candidates. Our results corroborate previous studies, such as those by Maher et al. [15] and Baig et al. [20], who also investigated the safety and therapeutic profile of withanolides isolated from *W. somnifera* (Solanaceae), a species widely used in traditional medicine and commonly recognized as a safe medicinal plant. Furthermore, our data are consistent with the study by Maciel et al. [32], who evaluated withanolides in adult zebrafish using the same dose range and similarly reported no signs of toxicity, which further reinforces the preclinical safety of this class of compounds and enables subsequent behavioral investigations.

**TABLE 1:** Results of acute toxicity tests (*n* = 6 fish/group).

Amostra	Mortality				96 h DL_50_ (mg/kg)/CI
	Control	D1	D2	D3	
Withanolide D	0	0	0	1	> 40

*Note*: Control: DMSO 3%; D1, Dose 1 (4 mg/kg); D2, Dose 2 (20 mg/kg); D3, Dose 3 (40 mg/kg); DL50—lethal dose to kill 50% of adult zebrafish; CI—confidence interval; 6 animals/group.

### 2.3 Locomotor Activity (Open Field Test)

The evaluation of zebrafish behavior is commonly based on parameters such as swimming speed, movement patterns, and overall locomotor activity, which represent essential indicators for assessing the effects of compounds on the central nervous system (CNS), particularly in studies focused on anxiety‐related disorders [33]. In this context, the open field test is a suitable approach, as it enables the detection of alterations in swimming behavior, including reduced locomotion or maintenance of normal activity, thereby reflecting the neurobehavioral effects of the tested substances [34].

**FIGURE 2:**
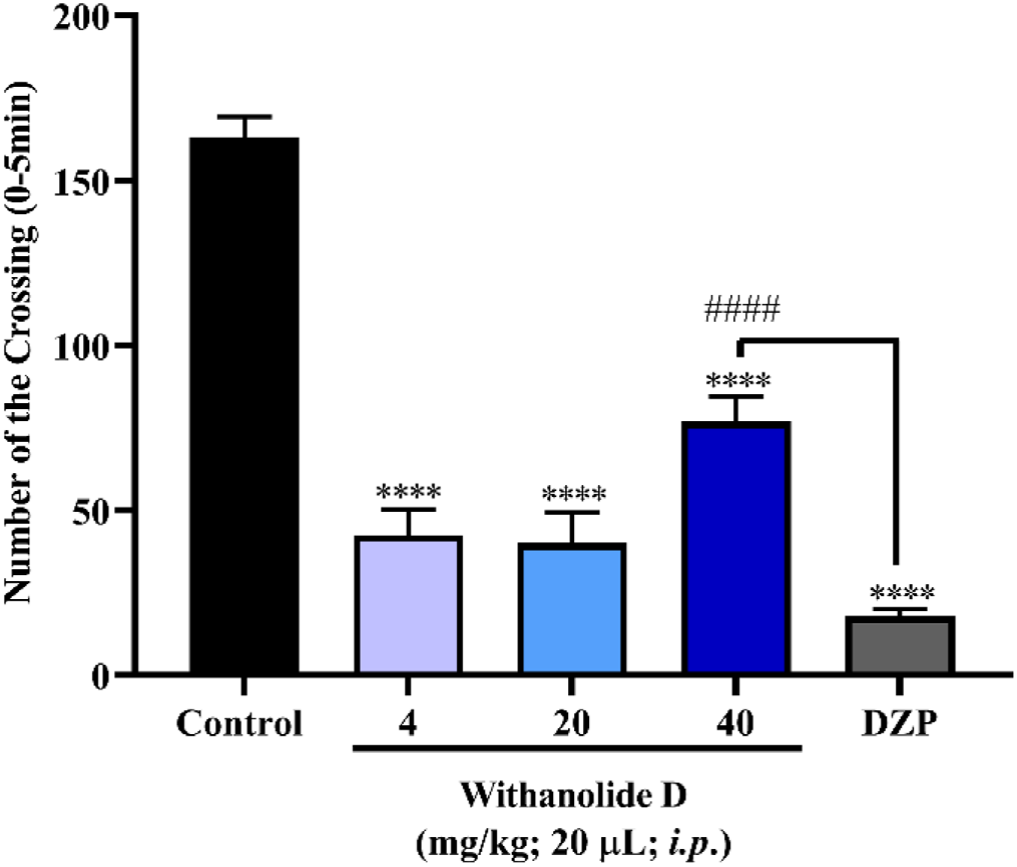
Effect of withanolide D on adult zebrafish locomotor behavior in the open field test (0–5 min). Values represent the mean ± standard error of the mean for 6 animals/group; ANOVA followed by Tukey's test. (****p < 0.0001 vs. control; ####*p* < 0.0001 vs. DZP).

In this study, one‐way analysis of variance (ANOVA) revealed that all tested doses of withanolide D (4, 20, and 40 mg/kg) significantly altered zebrafish locomotor activity (****p < 0.0001 vs. control), promoting a marked reduction in swimming behavior (Figure 2). This impairment is consistent with a sedative‐like profile, comparable to that induced by diazepam (DZP). Specifically, the 4 and 20 mg/kg doses produced effects similar to DZP, with the animals exhibiting lethargic behavior indicative of sedation. Although the 40 mg/kg dose also reduced locomotion, it did not promote a sedative state and also showed a statistically significant difference compared to DZP (####*p* < 0.0001 vs. DZP). Overall, these results indicate that withanolide D reduced locomotor activity at all tested doses, suggesting a sedative‐like effect and reinforcing its action on the CNS of adult zebrafish.

Comparable findings were reported by Kumar and Kalonia [35], who investigated the effects of *W. somnifera* root extract, rich in withanolides, in mice and observed anxiolytic activity accompanied by alterations in locomotor behavior, similarly associated with the effects of DZP. These results suggest that the observed sedative‐like profile may be related to modulation of the GABAergic system, which is a well‐established mechanism of action for DZP. Additionally, Candelario et al. [36], in a study using rat brain models, associated *W. somnifera* extracts with pharmacological activity against neurological disorders linked to dysfunction in GABAergic signaling, including anxiety‐related conditions.

### 2.4 Anxiolytic Assessment

**FIGURE 3:**
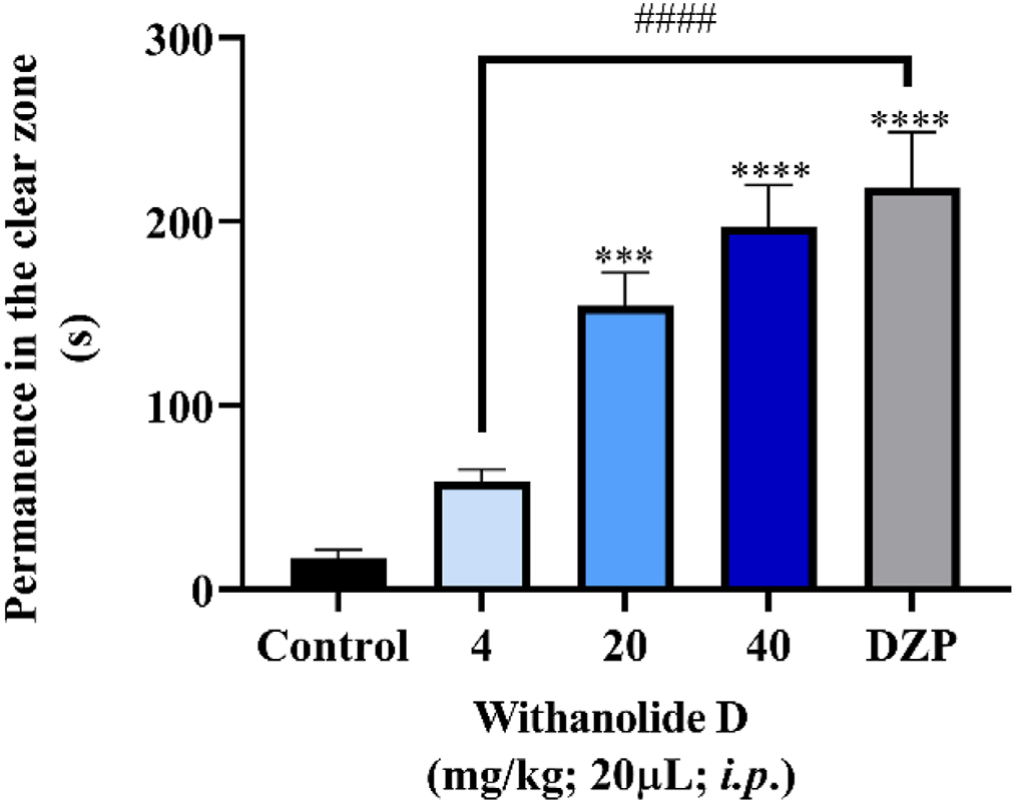
Effect of compounds and emulsions on the anxiety behavior of adult zebrafish in the light/dark test (0–5 min). Values represent the mean ± standard error of the mean for 6 animals/group; ANOVA followed by Tukey's test. (***p < 0.001, ****p < 0.0001 vs. control), and ####*p* < 0.0001 vs. diazepam (DZP).

The results obtained in the light/dark test (Figure 3) demonstrated that withanolide D, at doses of 20 and 40 mg/kg, significantly increased the time spent by zebrafish in the light compartment (***p < 0.001; ****p < 0.0001 vs. negative control – DMSO 3%, Figure 3), indicating a reduction in anxiety‐like behavior. This anxiolytic profile is consistent with the attenuation of the animals' natural preference for the dark zone, a behavioral response commonly associated with anxiety in zebrafish. Furthermore, the effects observed at these doses were comparable to those produced by the positive control diazepam (DZP), reinforcing the significant anxiolytic potential of the compound.

In contrast, the 4 mg/kg dose did not produce a similar anxiolytic effect and showed a significant difference compared to the DZP‐treated group (*p* < 0.0001 vs. DZP), suggesting that a higher dose is needed to trigger the anxiolytic response, with the minimum effective dose observed from 20 mg/kg. The significant difference between the 4 mg/kg group and the DZP group corroborates the presence of a dose‐dependent behavioral effect of withanolide D.

These findings are consistent with previous reports, such as the study by Zahiruddin et al. [10], which demonstrated the anxiolytic activity of withanolide class compounds isolated from *Withania somnifera*, a plant belonging to the same botanical family as the species investigated in the present study. Due to their natural origin and favorable safety profile, withanolides have been highlighted as promising candidates for the development of new therapeutic approaches for anxiety disorders.

Thus, to investigate the underlying mechanism of the anxiolytic effect of withanolide D, the 20 mg/kg dose, identified as the most effective concentration in the light/dark test, was selected for pharmacological neuromodulation analysis. Considering the involvement of the GABAergic system in anxiety‐related behaviors, the possible modulation of GABAA receptors was evaluated using flumazenil (FMZ), a selective antagonist of the benzodiazepine binding site. In general, FMZ is expected to block the effects mediated by GABAA receptors, preventing compounds that act through this pathway from exerting anxiolytic responses. Consequently, it is expected that zebrafish will maintain their natural anxiety‐like behavior.

**FIGURE 4:**
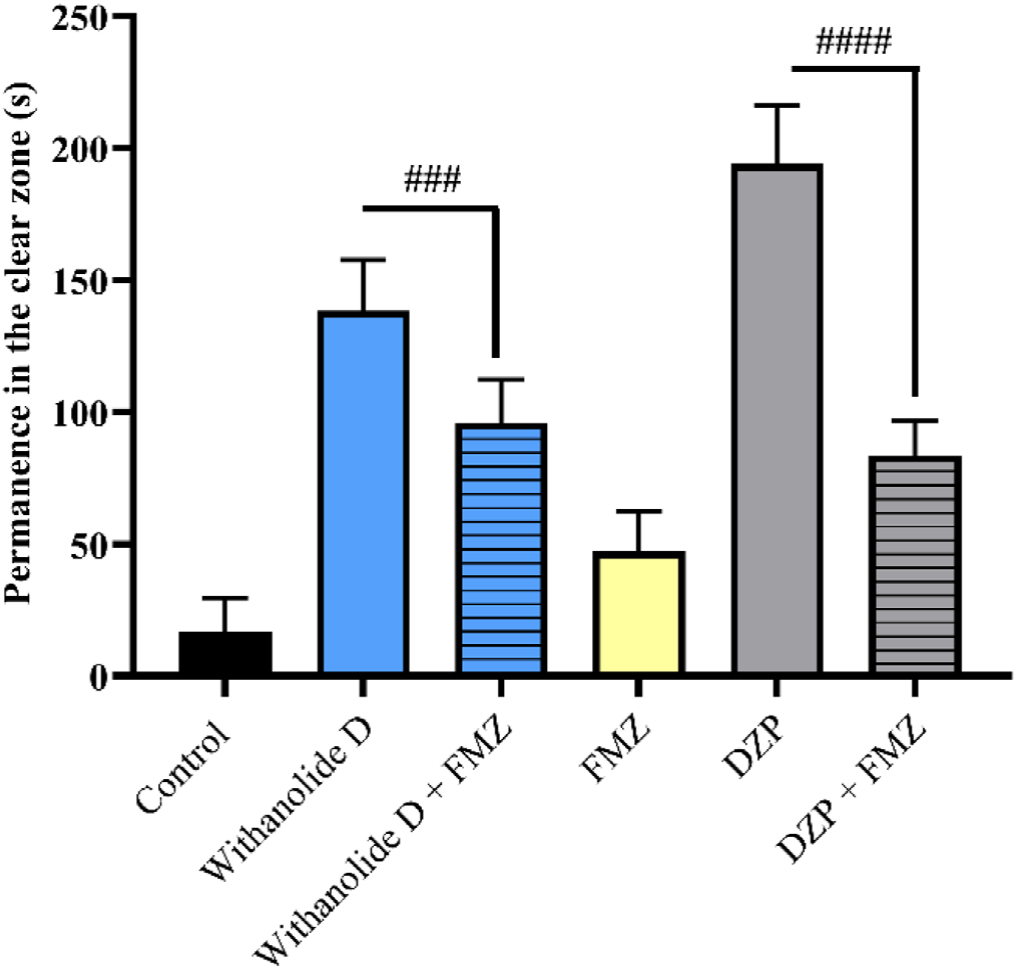
Anxiolytic mechanism of action via GABA of the sample withanolide D (20 mg/kg) in adult zebrafish in the light and dark test (0–5 min). Control—DMSO 3% (20 µL; i.p.). DZP (4 mg/kg; 20 µL; i.p.). The values represent the mean ± standard error of the mean (S.E.M.) for 6 animals/group. ANOVA followed by Tukey (###*p* < 0.001 vs. FMZ + withanolide D; ####*p* < 0.0001 vs. FMZ + DZP).

In this context, withanolide D (20 mg/kg; Figure 4) exhibited an anxiolytic effect that was significantly reversed by FMZ, as evidenced by the statistically significant difference between the group treated with withanolide D alone and the group treated with FMZ plus withanolide D (withanolide D vs. FMZ + withanolide D; Figure 4). These findings indicate that the anxiolytic response induced by withanolide D was blocked by FMZ, corroborating the involvement of the GABAergic system and supporting the participation of a benzodiazepine‐sensitive GABAA modulatory domain in its mechanism of action.

Taken together, these results suggest that withanolide D exerts its anxiolytic‐like effect through modulation of the GABAergic system. More specifically, the FMZ‐sensitive profile indicates that this effect is functionally associated with a benzodiazepine‐sensitive GABAA modulatory domain [21, 36, 50, 51, 54]. Previous studies have reported that compounds isolated from *W. somnifera*, including withanolides, can interact with GABAA channels and exhibit therapeutic potential in neurological disorders associated with impaired GABAergic signaling, such as anxiety and epilepsy [36]. Furthermore, Alex et al. [37] reinforced these findings by demonstrating significant antistress and anxiolytic activity of root and leaf extracts of W. somnifera in both animal models (rats) and humans, suggesting the involvement of the GABAergic pathway.

In addition, molecular docking analyses suggested favorable binding of withanolide D in the extracellular interface between the α1 and γ2 subunits of the GABAA receptor, a region functionally related to diazepam recognition [54]. However, the interaction pattern predicted for withanolide D differed from that observed for DZP, particularly by the predominance of hydrogen‐bond contacts and the absence of the characteristic hydrophobic interaction pattern described for classical benzodiazepines [52, 54]. Therefore, the present docking results do not support the conclusion that withanolide D occupies the classical benzodiazepine pocket in the same manner as diazepam. Instead, they are more consistent with a distinct binding orientation within, adjacent to, or functionally coupled to the same modulatory region [54, 57, 58]. Taken together, the behavioral reversal by flumazenil and the docking profile support the interpretation that withanolide D may act as a putative allosteric modulator of GABAA receptors, although the precise localization of its binding mode cannot be definitively established from static docking alone. Thus, the present in silico data should be interpreted as hypothesis‐generating evidence of a plausible receptor interaction site, rather than definitive proof of occupation of the classical benzodiazepine site [52, 54].

### 2.5 Inhibitory Avoidance Test

**FIGURE 5:**
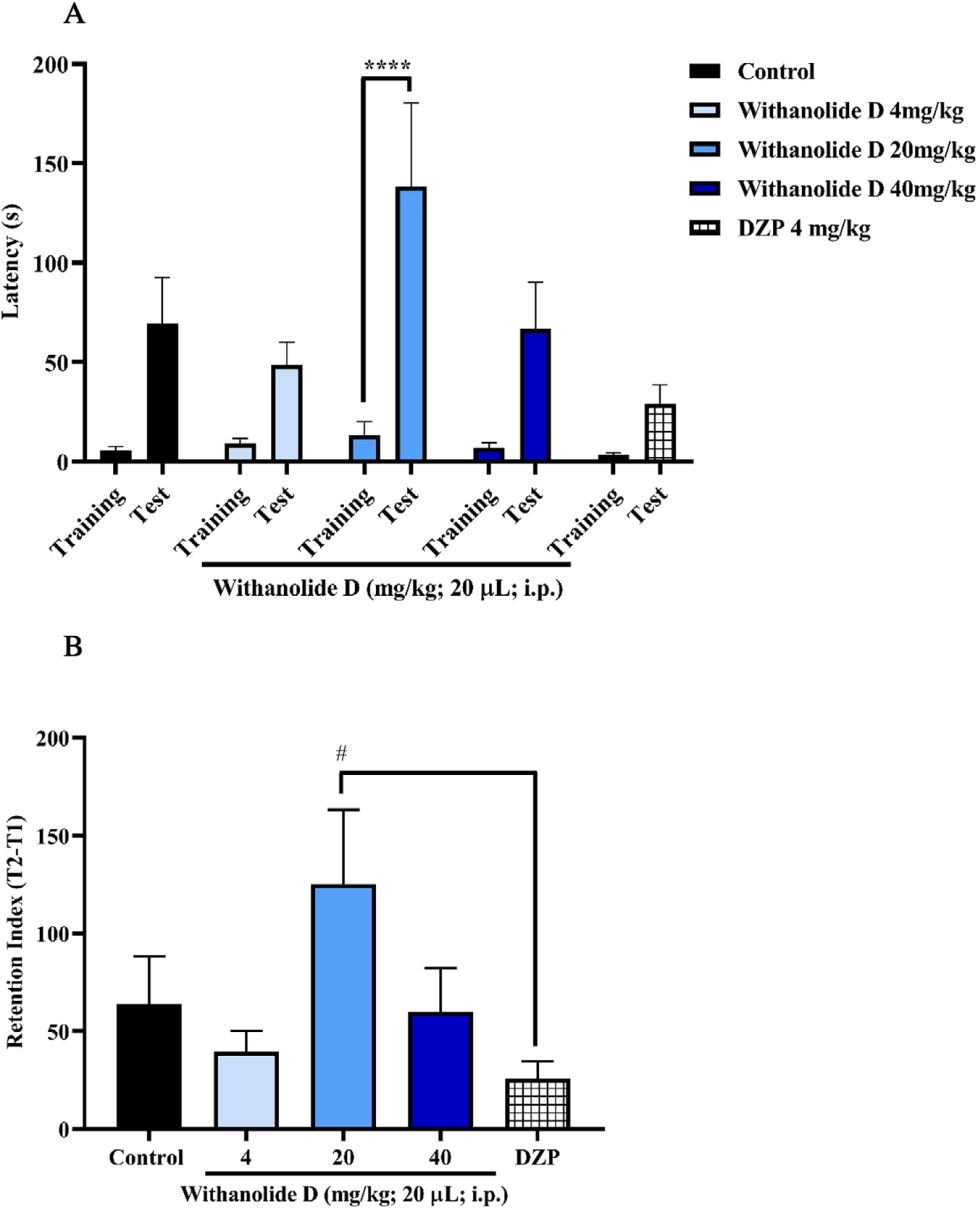
The evaluation of withanolide D on aversive memory in adult zebrafish studied the inhibitory avoidance task (0–5 min). (A) Latency to enter the dark area of the aquarium during training and test sessions. (B) Memory retention indices. The values represent the mean ± standard error of the mean for 6 animals/group; Two‐way ANOVA; One‐way ANOVA followed by Tukey's test (*p < 0.05;****p < 0.0001 vs. training and (#*p* < 0.05 vs. DNZP). Control—DMSO (3% dimethyl sulfoxide). DNZP—donepezil.

The inhibitory avoidance test, based on an aversive electroshock stimulus, demonstrated that withanolide D significantly enhanced memory retention in adult zebrafish. These findings indicate a positive effect on memory consolidation and retention, rather than on acquisition or retrieval processes. Significant effects were observed at two of the tested doses, particularly at 20 and 40 mg/kg (*p < 0.05; ****p < 0.0001 vs. test). Moreover, the memory retention index revealed no statistically significant differences between the withanolide D‐treated groups and the DNZP group, suggesting that zebrafish treated with withanolide D preserved memory of the aversive stimulus at levels comparable to those induced by the standard cognitive‐enhancing drug (Figure 5B). Although withanolides have been reported to exhibit cholinesterase inhibitory and antioxidant properties, these mechanisms were not specifically investigated in the present study. Therefore, the memory‐enhancing effect observed for withanolide D cannot be directly attributed to these pathways, but they may represent plausible mechanisms underlying its activity. However, the protective effect against ethanol‐induced memory impairment further supports a role of withanolide D in memory consolidation processes, although the precise molecular mechanisms involved remain to be elucidated.

Considering that withanolide D was administered prior to the training session and memory retention was assessed in a subsequent test session, the inhibitory avoidance paradigm used in this study primarily reflects effects on memory consolidation. The increased latency observed during the test session therefore suggests that withanolide D enhances consolidation of the aversive memory.

**FIGURE 6:**
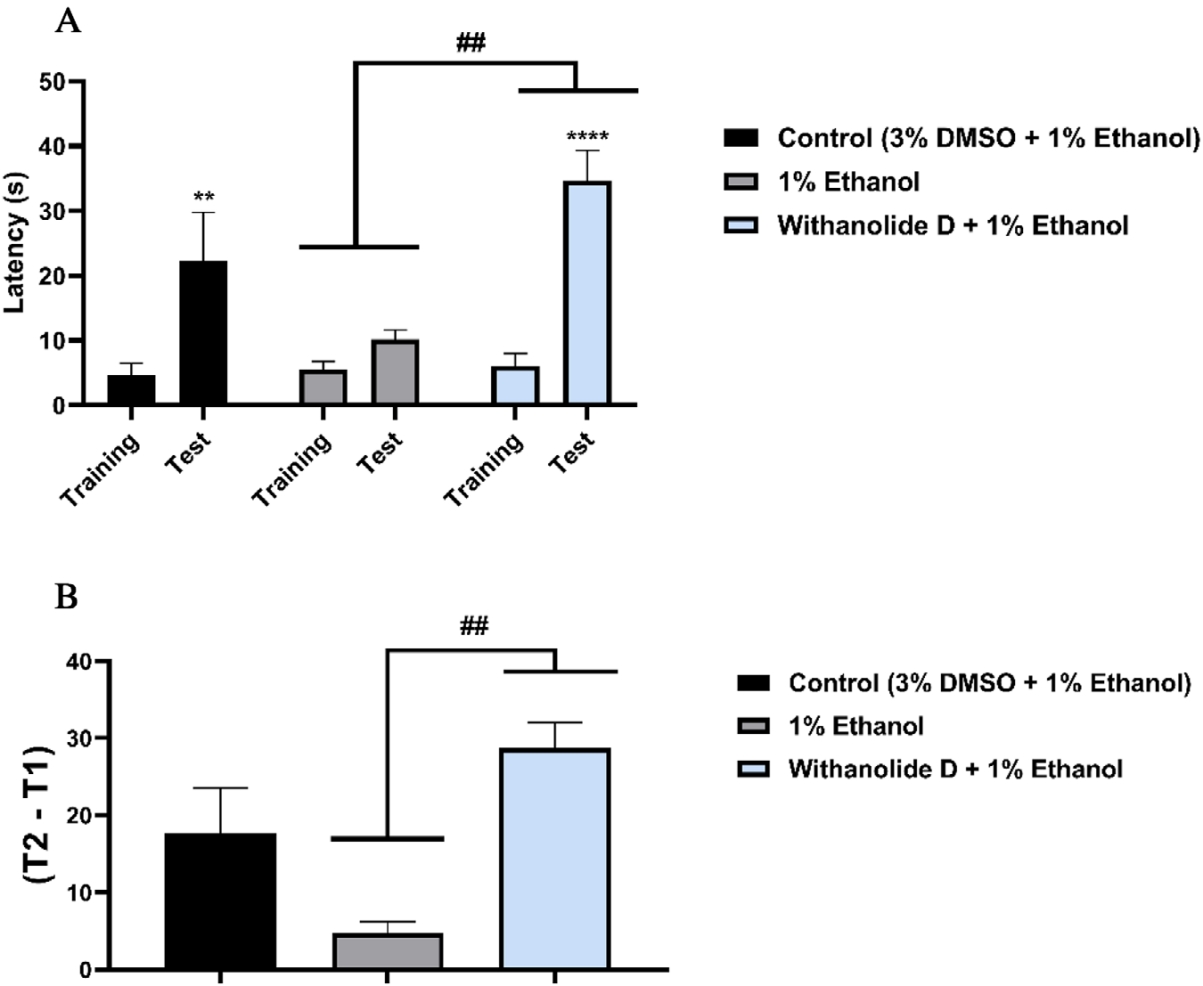
Inhibitory avoidance test. (A) The evaluation of withanolide D on aversive memory in zebrafish was studied using the inhibitory avoidance task after exposure to 1% ethanol (0–20 min). (B) Effect of pre‐treatment with withanolide D on the retention index of adult zebrafish memory after exposure to 1% ethanol (0–20 min). The values represent the mean ± standard error of the mean for 6 animals/group; Two‐way ANOVA; One‐way ANOVA followed by Tukey's test (##*p* < 0.01 vs. EtOH 1%) and unidirectional ANOVA followed by Tukey's test.

The effects of ethanol on memory acquisition in adult zebrafish were also investigated. Withanolide D, at the lowest effective dose (4 mg/kg), significantly preserved memory performance (****p < 0.0001 vs. test) and protected the animals against the deleterious effects of ethanol on memory acquisition (##*p* < 0.01 vs. ethanol) (Figure 6A).

Consistently, the memory retention index showed a significant difference between the group treated with withanolide D (4 mg/kg) and the group exposed to 1% EtOH (##*p* < 0.01 vs. EtOH), indicating that withanolide D effectively prevented ethanol‐induced memory impairment (Figure 6B).

Withanolide D is a relevant bioactive compound with promising therapeutic potential for the treatment of nervous system disorders. It has been extensively investigated in extracts obtained from the roots and other parts of *W. somnifera*, mainly due to its neuroprotective effects associated with acetylcholinesterase (AChE) inhibition and butyrylcholinesterase (BChE) inhibitory activity [7, 38, 39].

Moreover, in vivo studies exploring the multitarget potential of withanolides against mechanisms involved in beta‐amyloid (Aβ) processing and clearance have demonstrated that these compounds can increase the expression levels of two key Aβ‐degrading enzymes, insulin‐degrading enzyme (IDE) and neprilysin (NEP), particularly in brain regions selectively affected during Alzheimer's disease pathology [40, 41].

The brains of patients with Alzheimer's disease are characterized by the accumulation of Aβ plaques, which occur in substantially higher amounts than those observed in healthy individuals [42, 43]. Importantly, Aβ plaque burden is strongly correlated with the severity of cognitive impairment [44, 45]. Preclinical studies using mouse models have also supported the memory‐protective effects of *W. somnifera* [46, 47]. The neuroprotective properties of withanolides have been attributed, at least in part, to their antioxidant and anti‐inflammatory activities, which may contribute to the modulation of enzymes involved in Aβ degradation pathways [48, 49]. Therefore, the search for new cholinesterase inhibitors remains an important strategy for the development of drug candidates aimed at preventing Alzheimer's disease and related dementias.

### 2.6 Molecular Docking of Anxiolytic Assessment to the GABAergic System

In vivo behavioral results showed that withanolide D produced an anxiolytic effect similar to DZP, which was reversed by the presence of the antagonist FMZ. These findings indicate that the compound may act through modulation of the GABAergic system, functionally associated with a benzodiazepine‐sensitive GABAA modulatory domain, rather than allowing a definitive assignment of the classical diazepam‐binding mode. In this context, molecular docking simulations were utilized to investigate a plausible structural basis for the involvement of the GABAergic system [50, 51].

Predicting the binding sites of GABAA receptor‐modulating drug candidates is essential for the rational design of new anxiolytics and anticonvulsants. The structure of the GABAA receptor has multiple allosteric sites, which allows it to be modulated by various binding sites. Although the binding sites on this receptor may not be functionally equivalent, the DZP agonist is believed to interact with the α1‐D and γ2‐C chains in the receptor's extracellular domain, as described in structural studies of this receptor [54]. Molecular docking simulations showed that withanolide D displayed favorable predicted affinity (E(A) ≤ −6.0 kcal mol^−1^) [53] for the binding site located between the α1‐D and γ2‐C chains of the GABAA receptor, although it did not interact directly with amino acid residues located in the DZP binding site. Therefore, the docking results suggest interaction in a functionally related region, but not direct reproduction of the canonical diazepam interaction pattern.

**FIGURE 7:**
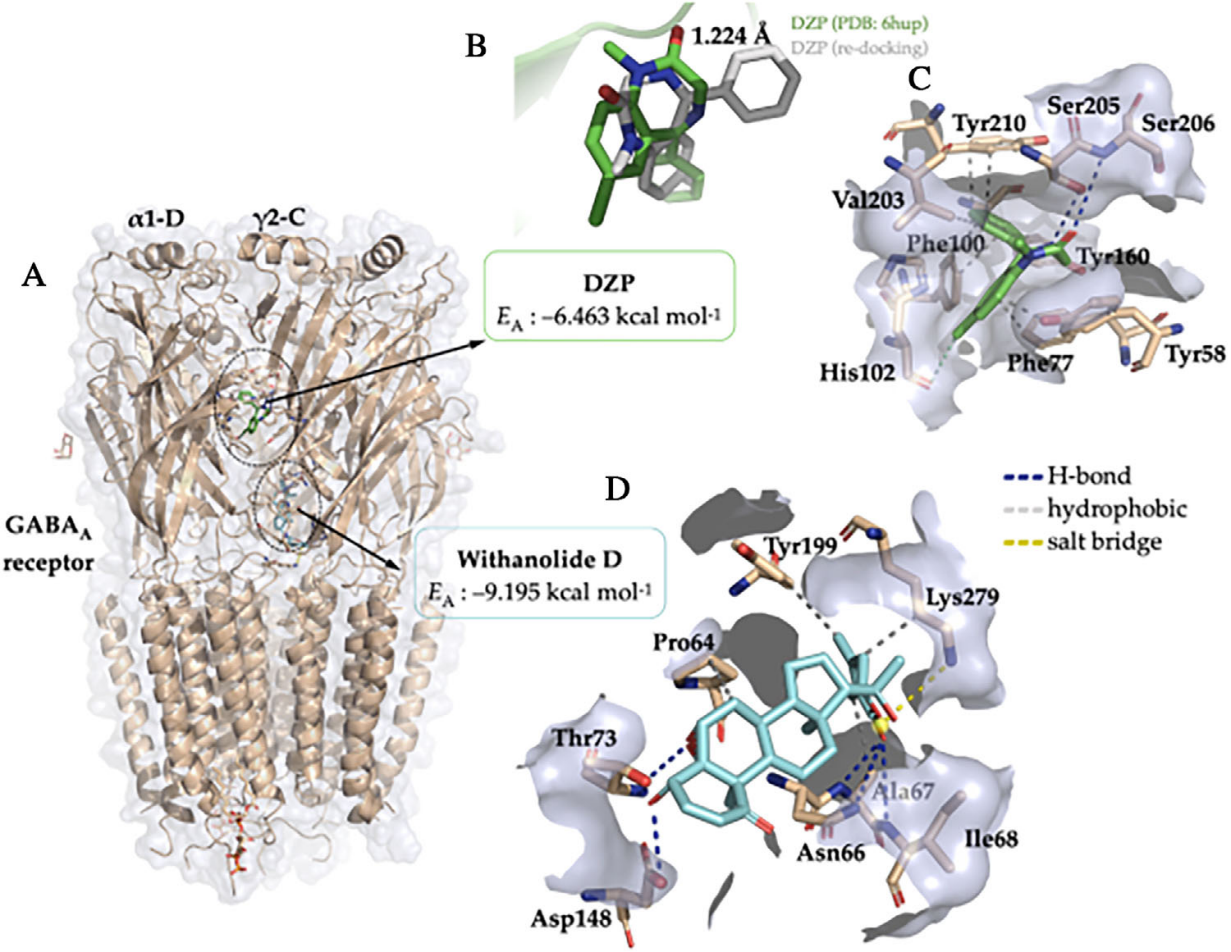
(A) Three‐dimensional (3D) visualization of the GABA_A_ receptor in the presence of the agonist DZP and the ligand withanolide D, located in the α1γ2 domain, (B) redocking do DZP (grey color) em relação a pose co‐cristalizada (green color), and 3D representation of the interactions of the ligands (C) DZP and (D) withanolide D in relation to the amino acid residues of the extracellular domain α1γ2 of the GABA_A_ receptor.

Based on the results, it was possible to observe that withanolide D binds with the GABAA receptor between the α1‐D and γ2‐C chains, where the co‐crystallized agonist DZP binds (Figure 7A), with an affinity energy (EA) in the order of −9.195 kcal mol^−1^ (Table 2). Within the accepted validation criterion formed by an RMSD of less than 2.0 Å [52], the EA values suggest that withanolide D has a greater predicted affinity for the binding site on the GABAA receptor when compared to DZP, which showed an EA calculated from the simulations in the order of −6.463 kcal mol^−1^ (Table 2) [53]. The DZP redocking simulation yielded an RMSD of 1.224 Å, indicative of the reproducibility of the simulation protocol with the agonist, exhibiting a low mean squared deviation from the co‐crystallized pose (Figure 7B). This result supports the suitability of the docking protocol for exploratory pose prediction in the selected receptor structure [52, 54]. However, predicted affinity values should not be interpreted as direct measures of pharmacological potency, especially when comparing chemically distinct ligands within a broad search space.

**TABLE 2:** Data from molecular docking simulations between the ligands DZP and withanolide D and the GABA_A_ receptor, expressed in affinity energy (*E*_A_) and root mean square deviation (RMSD), and details of ligand–protein interactions, expressed in interaction type, residue, and donor–acceptor distance.

Ligands	*E*_A_ (kcal mol^−1^)	RMSD (Å)	Interactions	
			Type	Residue (distance in Å)
Withanolide D	−9.195	1.635	Hydrophobic	Pro64C (3.96), Ala67C (3.89), Tyr199C (3.65), and Lys279D (3.66)
			H‐bond	Asn66C (3.18), Ala67C (2.16), Ile68C (3.18), Thr73C (2.15), and Asp148C (2.30)
			Salt bridge	Lys279D (4.31)
DZP*	−6.463	1.224	Hydrophobic	Tyr58C (3.52), Phe77C (3.47), Phe100D (3.83), Phe100D (3.92), Tyr160D (3.56), Val203D (3.95), Tyr210D (3.38), and Tyr210D (3.86)
			H‐bond	Ser205D (2.94) and Ser206D (3.16)
			Halogen bond	His102D (3.82)

*Note*: *Control ligand used as a comparison in molecular docking simulations.

DZP has a series of hydrophobic interactions with aromatic side‐chain amino acid residues, including Tyr58, Phe77, Phe100, Tyr160, and Tyr210 (Figure 7C), which characterize the hydrophobic binding cavity of benzodiazepines [52]. When analyzing the ligand–protein interactions, it was possible to observe that withanolide D formed H‐bond interactions with the polar portion of the residues Asn66 (3.18), Ala67 (2.16), Ile68 (3.18), Thr73 (2.15), and Asp148 (2.30) (Figure 7D), where donor‐acceptor distances of approximately 2.1 Å reveal strong H‐bond interactions (with residues Ala67 and Thr73), while donor‐acceptor distances > 2.5 Å indicate weaker H‐bond interactions (Table 2) [53]. In addition, the alkene located in the cyclic ester structure of withanolide D contributes to the formation of hydrophobic interactions with the aromatic portion of Tyr199 and with the alkyl portion of Lys279 (Figure 7D), indicating that unsaturation can favor the formation of hydrophobic interactions with the receptor [54, 55]. However, the interaction pattern predicted for withanolide D differed from that observed for DZP, particularly by the predominance of H‐bond contacts and the absence of the characteristic hydrophobic interaction pattern described for classical benzodiazepines [52, 54].

At the end of the cycle of independent molecular docking simulations, it is noteworthy that the compound exhibits interactions with amino acid residues located in proximity to those that directly interact with DZP. These interactions occur within the loop formed between the α1 and γ2 subunits, facilitated by weak van der Waals interactions, which can be expressed as hydrophobic interactions. Taken together, these findings support the interpretation that withanolide D may bind within, adjacent to, or functionally coupled to the same modulatory region associated with DZP recognition, rather than occupying the classical benzodiazepine pocket in the same manner as diazepam [54, 57, 58]. This observation lends support to the hypothesis that the anxiolytic effect exhibited by the agonist and withanolide D is attributable to functionally related, although not necessarily identical, modulatory mechanisms.

The grid box was adjusted to 94 × 92 × 126 Å to cover the whole GABAA receptor structure to find alternative binding sites in the GABAergic system [56, 57]. A substantial amount of research has emerged that establishes a correlation between in vitro experimentation with the GABAergic system and molecular simulation. These studies have led to the identification of an alternative binding region. The region in question has been localized within the loop region of the β1 and β2 subunits [58]. Thus, the present docking strategy was designed as a receptor‐wide exploratory analysis rather than a narrowly restricted binding‐pocket refinement. This approach provides insight into the allosteric modulation of GABAA receptors, corroborating the similar anxiolytic potential between withanolide D and DZP observed in the in vivo assay with zebrafish. Nevertheless, because the present analysis is based on static docking, without post‐preparation receptor energy minimization or dynamic receptor‐ligand evaluation, the proposed binding mode should be interpreted as hypothesis‐generating evidence of a plausible interaction site, rather than definitive proof of occupation of the classical benzodiazepine site.

### 2.7 ADMET Study

#### 2.7.1 Lipophilicity Potential (LP) and Structural Complexity

**FIGURE 8:**
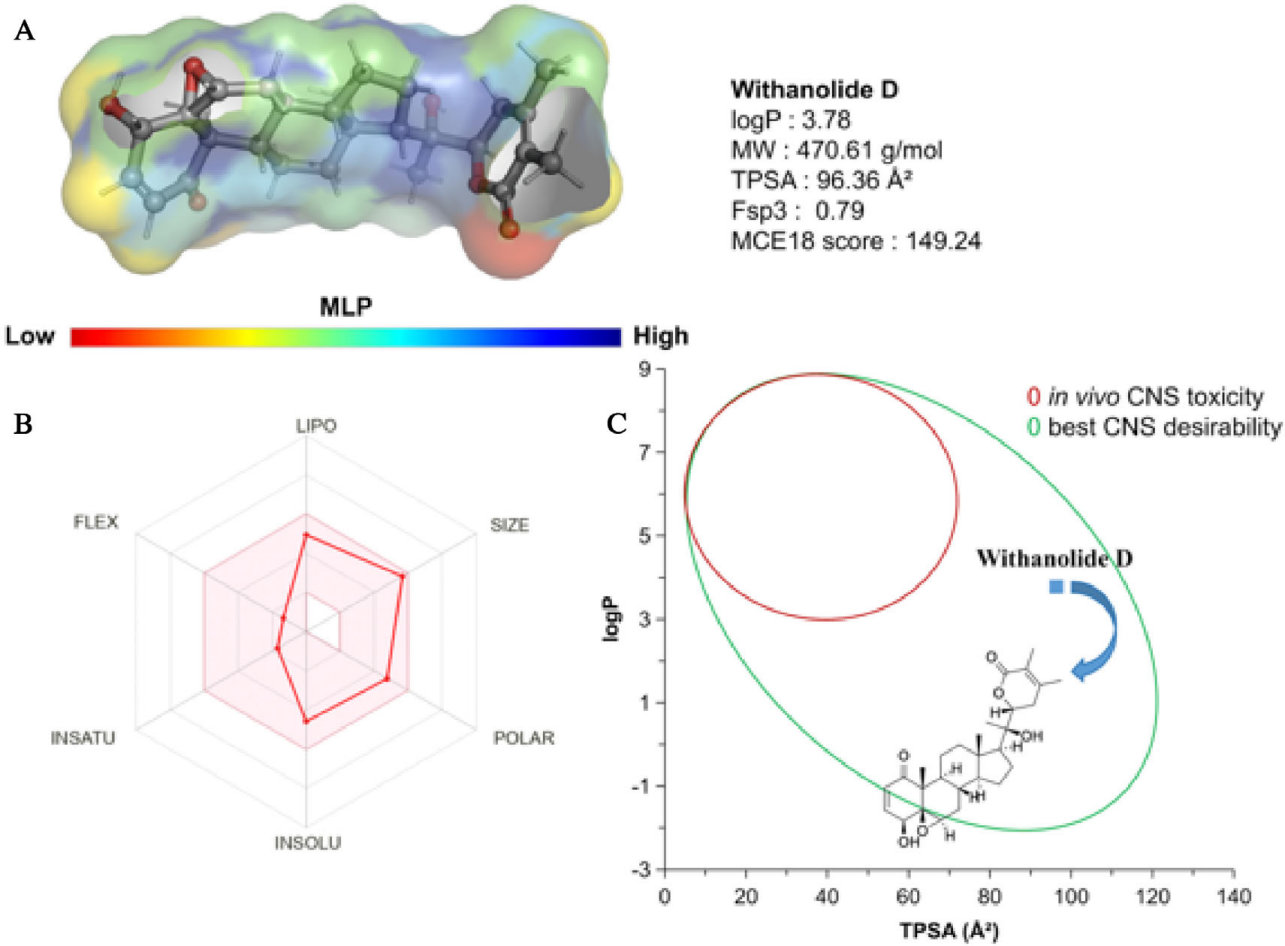
(A) Map of molecular lipophilicity (LP) potential, (B) oral bioavailability radar with the main attributes that affect the pharmacokinetics of withanolide D: lipo (logP), size (MW), polar (TPSA), insolubility, insatu (Fsp3), and flexibility, and (C) alignment between logP and TPSA to estimate the CNS desirability profile.

When analyzing the molecular lipophilicity potential (LP), it was possible to note that withanolide D has a polycyclic structure rich in Fsp^3^ (0.79) which enables the formation of an essentially hydrophobic molecular surface (green to blue spectra in Figure 8A), with logP calculated at 3.78, within a lipophilicity threshold of between 3.0 and 5.0 for more lipophilic compounds with CNS activity. The MCE18 score of around 149.24 is indicative of structural complexity with a very good alignment between degree of structural novelty and synthetic accessibility, basically following the new trends observed in patent‐registered compounds in recent years, such as high lipophilicity and high polarity [59].

In terms of polarity, it can be seen that the lactone‐derived rings (OC═O) and the hydroxyl groups (OH) contribute strongly to the formation of the water‐soluble polar portion (low MLP), with TPSA calculated at 96.36 Å(2) (Figure 8A). These descriptors are aligned within a favorable threshold attributed to drug‐like compounds with viable bioavailability for achieving the therapeutic effect (Figure 8B). When the lipophilicity (logP) and polarity (TPSA) descriptors were aligned, it was possible to observe that the compound resides in a physical chemical space formed by CNS‐active compounds with a low toxic response in vivo (Figure 8C), according to the Pfizer, Inc. biopharmaceutical classification system (TPSA 75‐120 Å2) [60, 61].

#### 2.7.2 Cell Effective Permeability Prediction

According to the Pfizer, Inc. pharmacokinetic parameter classification system, compounds with high lipophilicity can readily permeate more selective biological membranes, such as the Madin–Darby Canine Kidney (MDCK) cell line, used to measure the permeability of candidates with CNS action. The *P*_app_ values observed in this context are typically in the order of ×10^−5^ cm/s, indicating a promising effect on the CNS [61, 62]. When the physicochemical descriptors of withanolide D were aligned, it was possible to estimate a pharmacokinetic profile based on high permeability in more selective cell membranes (BBB‐like), favoring the compound's anxiolytic effect.

**FIGURE 9:**
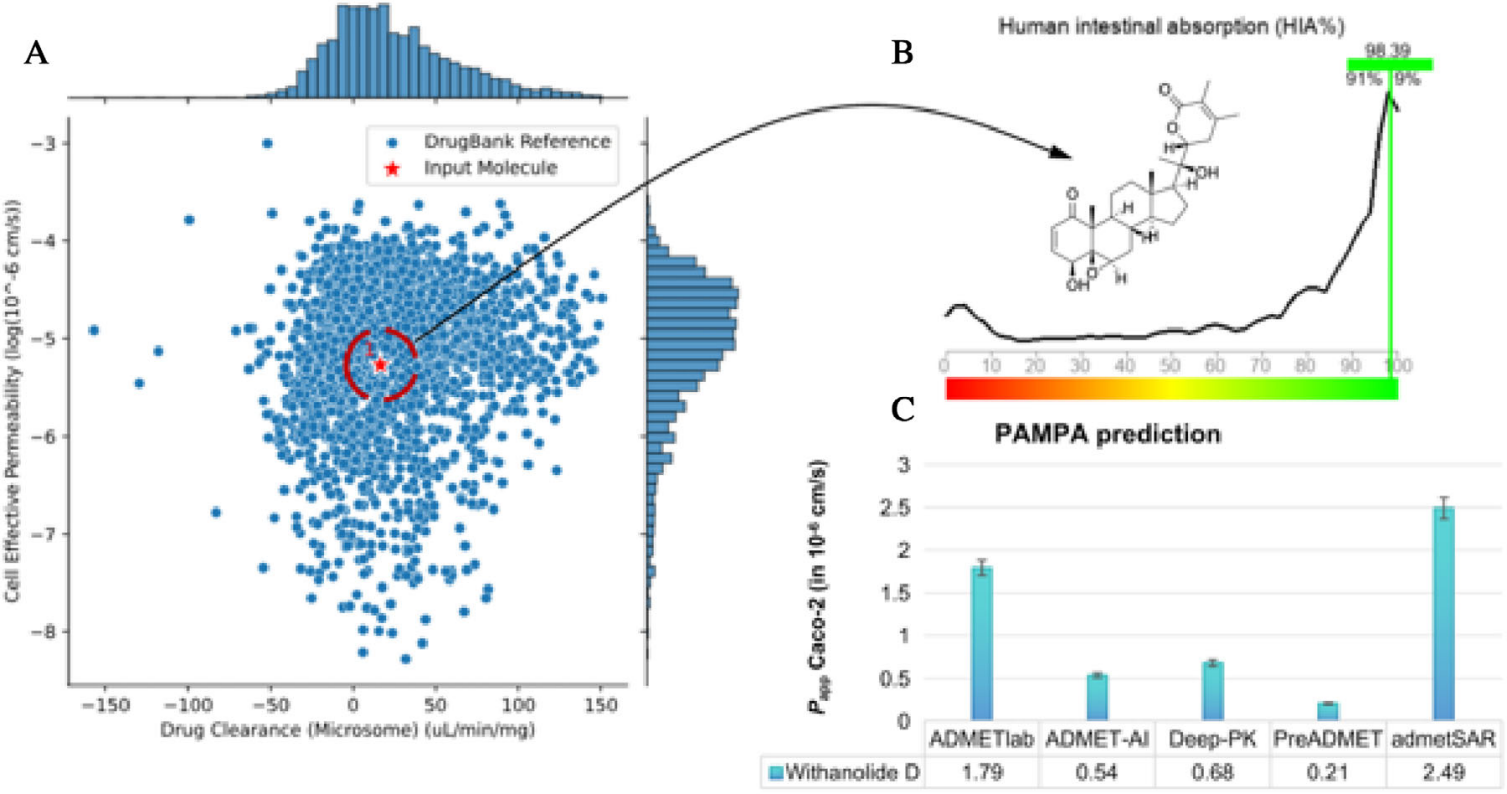
(A) Prediction of PAMPA descriptors guided by artificial intelligence for prediction of the spectrum of cell effective permeability (log *P*_app_) and drug clearance in microsome system, (B) structure‐based human intestinal absorption (HIA) prediction, and (C) prediction of passive cell permeability (*P*_app_) in Caco‐2 cells.

From an absorption, distribution, metabolism, excretion, and toxicity (ADMET) profile prediction guided by artificial intelligence, it was possible to observe that withanolide D presents a structural similarity with compounds with low hepatic clearance and moderate cell permeability, deposited in the Drugbank database, within a threshold formed by −6.0< logP(app) < −5.0 (Figure 9A) [63], which corroborates with the estimated human intestinal absorption (HIA) of at least 98% (Figure 9B), showing a similarity of data with at least 91% of the molecular fragments with optimized HIA [64].

With the consensus prediction of ADMET, it was possible to observe that most of the estimated values of *P*_app_ Caco‐2, in different predictive models, are concentrated in a range of 0.2–0.7 × 10^−6^ cm/s, indicating that the compound can diffuse at a moderate speed in intestinal cell membranes, ensuring gradual absorption (Figure 9C) [65]. On the other hand, a *P*_app_ MDCK value of around 2.73 × 10^−5^ cm/s is estimated, indicating that withanolide D shows better passive diffusion in more selective biological membranes, including the blood–brain barrier (BBB), reinforcing intestinal absorption, and favoring activity in the CNS (Table 3).

**TABLE 3:** ADMET properties expressed in PAMPA descriptors, cytochrome P450 (CYP450) isoform‐dependent metabolism and predicted toxicity for withanolide D.

Properties	Values	Sources
*P*_app_ MDCK	2.73 × 10^−5^ cm/s	ADMETlab 3.0
P‐gp efflux	—	ADMETlab 3.0
*Cl*_int,u_	6.92 mL/min/kg	ADMETlab 3.0
*Cl*_Micro_	16.64 µL/min/mg	ADMET‐AI
*Cl*_Hepa_	83.91 µL/min/10^6^ cells	ADMET‐AI
*V*_dss_	0.84 L/kg	ADMETlab 3.0
CYP2C9	0.01	ADMET‐AI
CYP2D6	0.02	ADMET‐AI
CYP3A4	0.69	ADMET‐AI
HLM stability	+++	ADMETlab 3.0
Ames mutagenicity	0.61	ADMETlab 3.0
H‐HT	0.67	ADMETlab 3.0

*Note*: For the classification endpoints, the prediction probability values are transformed into six symbols: 0–0.1 (—), 0.1–0.3 (–), 0.3–0.5 (‐), 0.5–0.7 (+), 0.7–0.9 (++), and 0.9–1.0 (+++).

Abbreviations: *P*_app_ MDCK, passive permeability in Madin–Darby canine kidney cell line; P‐gp, P‐glycoprotein; *Cl*_int,u_, intrinsic hepatic clearance; *Cl*_Micro_, clearance in liver microsomes; *Cl*_Hepa_, clearance in human hepatocytes; *V*_dss_, volume of distribution of steady state; HLM, human liver microsome; H‐HT, human hepatotoxicity.

Furthermore, the compound is not a P‐gp substrate, avoiding passive efflux back into the lumen of the gastrointestinal tract, while the predicted intrinsic clearance (*Cl*_int,u_) of 6.92 mL/min/kg indicates a low clearance of the biotransformed molecular fraction in the human liver, preserving good oral bioavailability (Table 3).

#### 2.7.3 Predicting Oral Bioavailability

A consensus prediction across ADMET multiplatforms revealed a correlation between *P*_app_ Caco‐2 rates, clearance, and intestinal absorption on the oral bioavailability of withanolide D [66, 67]. The prevailing consensus among experts in the field suggests that the compound in question exhibits a predominantly lipophilic character, as evidenced by its moderately elevated lipophilicity levels (logP > 3.0), aligned with low aqueous solubility. This property has the potential to enhance passive diffusion within the lumen of the gastrointestinal tract, a phenomenon that may be attributed to increased permeability. This finding aligns with the predicted *P*_app_ Caco‐2 rates (Caco‐2 permeability) of > −5.3 (log cm/s), indicating a convergence of the *P*_app_ values to > 5.0 × 10^−6^ cm/s (see Table 4). Furthermore, the predicted *P*_app_ Caco‐2 value of approximately 21.56 nm/s aligns with *P*_app_ ranges greater than 2.1 × 10^−6^ cm/s, as classified by the biopharmaceutical classification system [66]. This is consistent with predicted intestinal (human) absorption rates of over 90%, as indicated in Table 4. This finding supports the HIA prediction made by the data similarity test (Figure 9B). The pkCSM estimate in Caco‐2 permeability (log *P*_app_ in cm/s) has been shown to converge to *P*_app_ rates > 8 × 10^−6^ cm/s for log *P*_app_ values tending to 0.90, thereby estimating a moderate permeability for withanolide D (log *P*_app_ = 0.83).

**TABLE 4:** Resultados da predição consensual multiplataforma de ADMET para o withanolide D.

Datasets	Lipophilicity (logP)	Caco‐2 permeability	Intestinal absorption (human)	P‐gp inhibitor	Clearance	BBB
ADMETlab 3.0	3.12	−4.747	(—)	(—)	6.92 mL/min/kg	(+)
ADMETboost	3.5	−5.31	70.69%	39.86%	41.48 mL/min/g	19.34%
ADMET‐AI	3.50	−5.27	1.00	0.69	16.64 µL/min/mg	0.48
PreADMET	2.95	21.56 nm/s	94.73%	Inhibitor	—	0.29 (*C*_brain_/*C*_blood_)
pkCSM	3.49	0.83	99.2%	Yes	0.35 log mL/min/kg	−0.32 (logBB)
admetSAR 3.0	3.5	−4.6	91.0%	55.8%	—	1.00

Dataset	PPB	*V*_dss_ (human)	CYP2C9	CYP2D6	CYP3A4	DILI
ADMETlab 3.0	79.8%	0.83 L/kg	(+++)	(+++)	(+++)	0.29
ADMETboost	52.22%	4.10 L/kg	30.18%	54.26%	35.66%	42.7%
ADMET‐AI	88.52%	—	0.01	0.02	0.69	0.32
PreADMET	93.65%	—	—	Non‐substrate	Substrate	—
pkCSM	—	0.89 L/kg	—	No	Yes	No
admetSAR 3.0	81.39%	0.51 L/kg	33.0%	24.9%	92.9%	73.6%

When these values are aligned with the estimated low intrinsic clearance (6.92 mL/min/kg), an alignment can be observed between high permeability and low microsomal clearance. This, in turn, leads to high oral bioavailability after first‐pass metabolism (clearance < 8.0 mL/min/kg) [62]. The ADMET‐AI web service employs a classification system from AstraZeneca, which is deposited in the ChEMBL database. Values within an optimal clearance range, defined as 3.0–150 mL/min/g, are concentrated in this system. It is noteworthy that withanolide D converges to the ideal spectrum with an estimated liver microsome clearance of 16.64 µL/min/mg (Table 4) [68, 69]. The results suggest that the compound may exhibit high bioavailability in the systemic circulation.

#### 2.7.4 Plasma Protein Binding

The estimation of plasma protein binding (PPB) demonstrates its contribution to systemic distribution and drug access to different physiological environments, such as cell membranes. The degree of binding of these serum proteins directly correlates with the reduced permeability of the molecules, which can potentially impact the permeability of the BBB [9]. In this study, the prediction of PPB rates > 79% was observed for the majority of the analyzed databases (Table 4), indicating that the bioavailable molecular volume may be more confined to the blood. This suggests that a small free molecular fraction in the plasma has BBB permeability. In this instance, a BBB permeability (logBB) of approximately −0.32 is predicted, expressing a moderate passive diffusion (see Table 4). This finding corroborates the predicted blood–brain distribution coefficient (*C*_brain_/*C*_blood_) of 0.29 [70]. The classification system indicates that logBB values ranging from −1.0 to 0.3 and BB (brain/blood) values between 0.1 and 2.0 are associated with moderate distribution ranges to the central nervous system (CNS). These results suggest that withanolide D presents a free molecular fraction in blood plasma that can access the CNS via BBB permeability.

The degree of confidence in the results may vary depending on differences in units of measurement, algorithms, and the applicability domains of the platforms used; therefore, the data should be interpreted qualitatively in the context of ADMET in silico predictions.

#### 2.7.5 Volume of Distribution

The hydrophobic nature of withanolide D is closely related to its human volume of distribution of steady state (Vdss), which expresses the theoretical volume of the total drug dose capable of uniformly distributing between blood plasma and tissues [9]. According to the findings of the ADMET consensus test, the logP rates are predicted to be greater than 3.0. This suggests that the Vdss rates are likely to be less than 0.9 L/kg, as indicated by most algorithms. This indicates that the molecular fraction of bioavailability in systemic circulation has a higher affinity for blood plasma and minimal distribution to biological tissues, such as adipose tissue and the BBB. This suggests that a pharmacological active principle may act in the CNS, with control of the daily oral dose administered. However, the actual efficacy of the compound in the CNS for the treatment of anxiety disorders can only be corroborated in in vivo assays.

#### 2.7.6 Predicting Metabolism Profile

The consensus ADMET prediction indicated that the metabolism profile may be directly associated with the efficiency of withanolide in systemic circulation and the safety profile after oral administration [71]. The consensus test demonstrated that the compound exhibited low susceptibility to being a substrate for isoforms such as CYP2C9 and CYP2D6 in the human liver microsome (HLM) system. However, it demonstrated structural susceptibility to being a substrate for the CYP3A4 isoform, which is predominant in the human liver for xenobiotic metabolism. This substrate may undergo aliphatic hydroxylation in its isolated unsaturated centers [72]. However, the prediction of drug‐induced liver damage (DILI) may be inconclusive regarding the metabolic processes and toxicity of metabolites formed in first‐pass metabolism. This may be a limitation in the predictive model when analyses extend to toxicity endpoints. Given the inherent discrepancies in the methodological approaches employed by the disparate data platforms (como o ADMETboost), a direct and objective comparative analysis of the absolute values is rendered unfeasible. Consequently, the interpretation of the results was conducted qualitatively, with a focus on identifying concordant trends across the datasets.

#### 2.7.7 Site of Metabolism and Toxicity Prediction

Phase I metabolism, mediated by CYP450 isoenzymes, can result in the generation of reactive secondary metabolites, often formed by processes such as epoxidation, generated from the hydroxylation of aromatic centers [73, 74]. These substructures can give rise to metabolites capable of binding covalently to proteins and DNA, causing liver damage. In addition, these biotransformation reactions can significantly impact the hepatic clearance pathways, both in hepatic microsomes and hepatocytes, affecting oral bioavailability. In this predictive test, it was possible to observe that the compound can be metabolized by the CYP3A4 isoform, forming epoxide‐based metabolites that are reactive in hepatic microsomes and can be slowly released in cellular hepatocytes, which can result in a toxic response due to metabolic activation.

**FIGURE 10:**
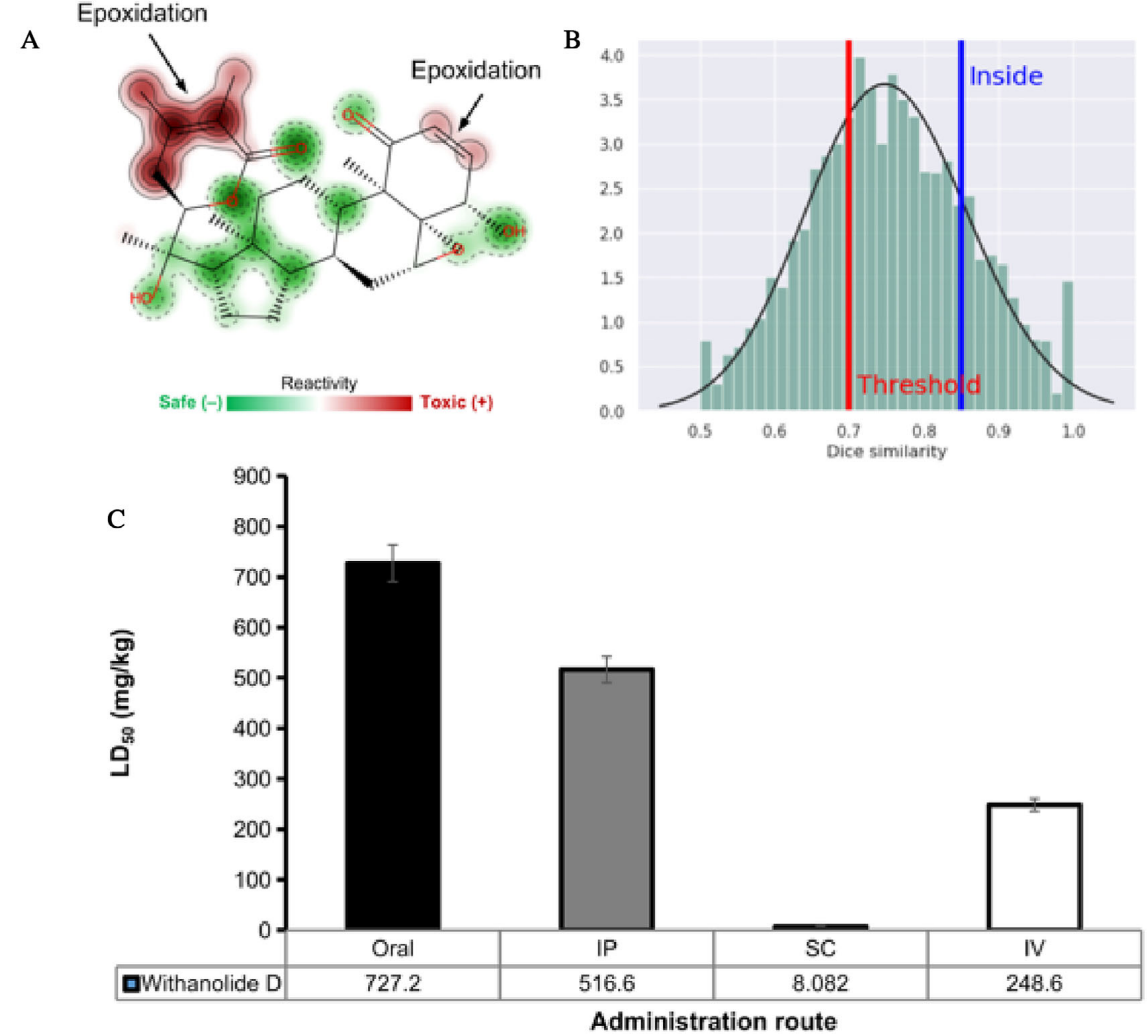
(A) Prediction of the site of metabolism and reactivity, (B) similarity index of the test for identifying toxic molecular fragments, and (C) prediction of lethal dose (LD_50_) in different routes of administration (oral, intraperitoneal—IP, subcutaneous—SC, and intravenous—IV).

From the site of metabolism prediction, it was possible to observe that withanolide D has two aliphatic hydroxylation‐dependent epoxidation sites, located at the ketone ring site (Figure 10A), catalyzed by the CYP3A4 isoform in phase I metabolism, with a data similarity > 0.8 in relation to toxic and reactive fragments (Figure 10B) [72]. This fragment has often been related to mutagenic compounds, since the epoxide is an electrophilic receptor capable of interacting covalently with nucleophiles present in DNA [73]. This result corroborates the toxicity prediction, where a probability of at least 0.6 of the compound inducing mutagenic or hepatotoxic damage was estimated (Table 3). However, it should be noted that the organic toxic response is a typical behaviour observed in in vivo assays. Therefore, experimental validation is needed for corroboration.

In addition, these are biotransformation processes that can affect the daily oral dose administered of the compound [74]. In the prediction of acute toxicity in rats, a lethal dose (LD_50_) of around 727.2 mg/kg was estimated for the oral route of administration, which is below the ideal expected for a well‐tolerated toxic response, indicating an organic toxic response due to metabolic activation [75]. In addition, LD_50_values of less than 600 mg/kg were estimated for the other routes of administration, which include intraperitoneal (IP), subcutaneous (SC) and intravenous (IV), indicating that the safest route of administration is oral, despite the predicted toxic response (Figure 10C).

#### 2.7.8 Molecular Dynamics

**FIGURE 11:**
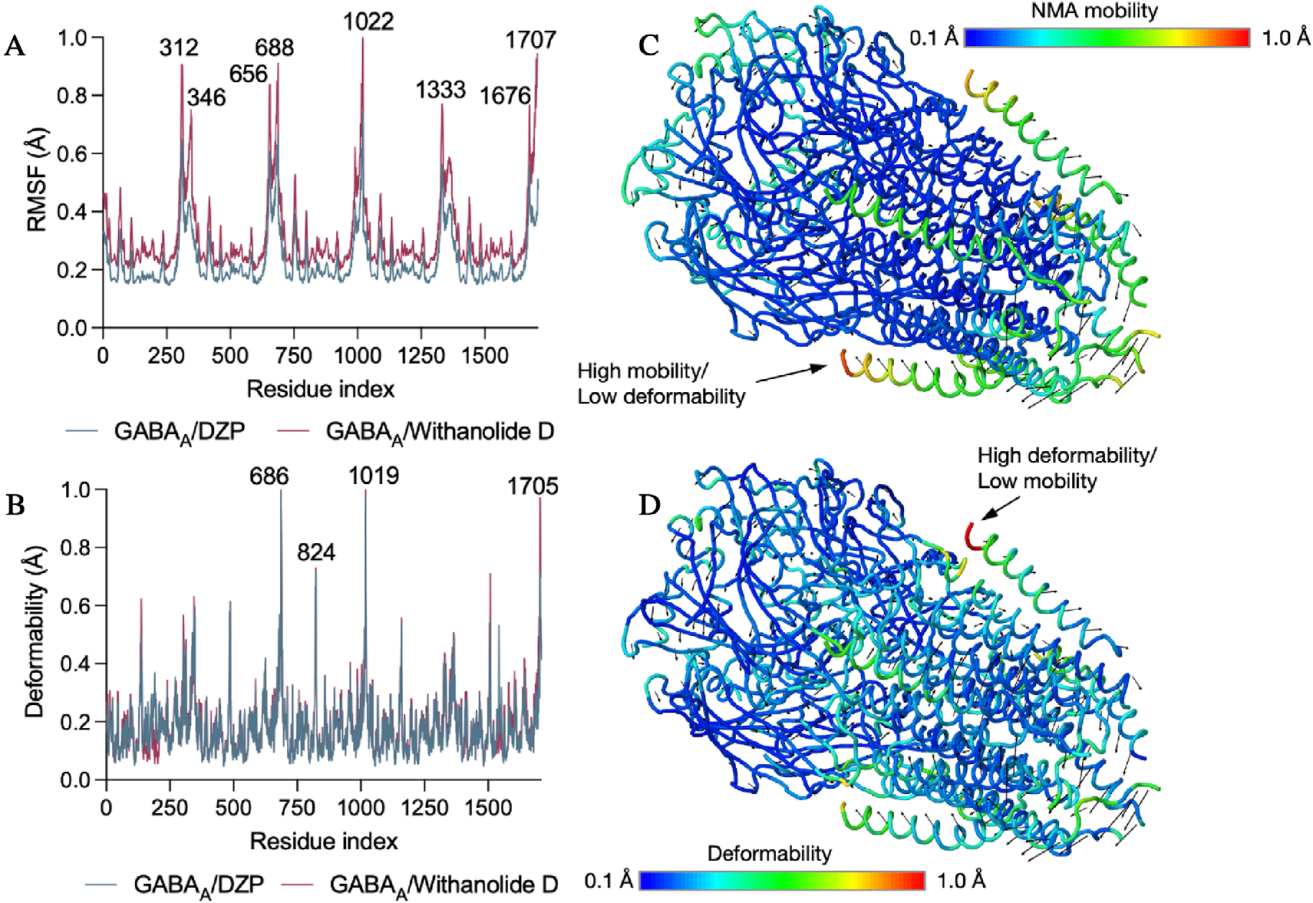
Comparative dynamics of the GABA_A_ receptor complexed with diazepam (blue) and withanolide D (pink). (A) RMSF profile per residue, highlighting the increase in global mobility induced by withanolide D. (B) Deformability index indicating the hinge regions of the structure. (C) Mapping of NMA mobility on the 3D structure, with a color scale ranging from low (blue) to high (red) mobility. (D) Mapping of high deformability regions, highlighting the mechanical pivots responsible for the conformational transitions.

The Normal Mode Analysis (NMA) module was employed to observe the impact of withanolide D on the natural dynamics of the GABA_A_ receptor, as expressed in the 6HUP PDB. The results demonstrated fluctuations in amino acid residues, considering the mobility (B‐factors in Å) and deformability of the C‐terminal, N‐terminal, and Cα structures. In this round of molecular dynamics simulations, considerable fluctuations were observed in withanolide D around residues 321 (A‐chain), 656–688 (B‐chain), 1022 (C‐chain), and 1707 (E‐chain), with root mean square fluctuations (RMSF) > 0.8 Å (Figure 8A). It is noteworthy that the binding of withanolide D resulted in an enhancement of sensitivity in GABA_A_ channels, attributable to the high mobility that ensued from the fluctuations. The DZP agonist exhibited its maximum fluctuation peak (RMSF) at residue 1022, with an RMSF of approximately 0.72 Å, whereas the highest fluctuation observed for withanolide D demonstrated an RMSF of 0.98 Å at the corresponding residue (Figure 11A). Withanolide D promotes a greater range of motion (RMSF) in the GABA_A_ receptor compared to Diazepam, but the structural deformation centers remain localized in specific residues (686, 1019, and 1705), which act as focal points for the conformational transition of the receptor (Figure 11B).

**FIGURE 12:**
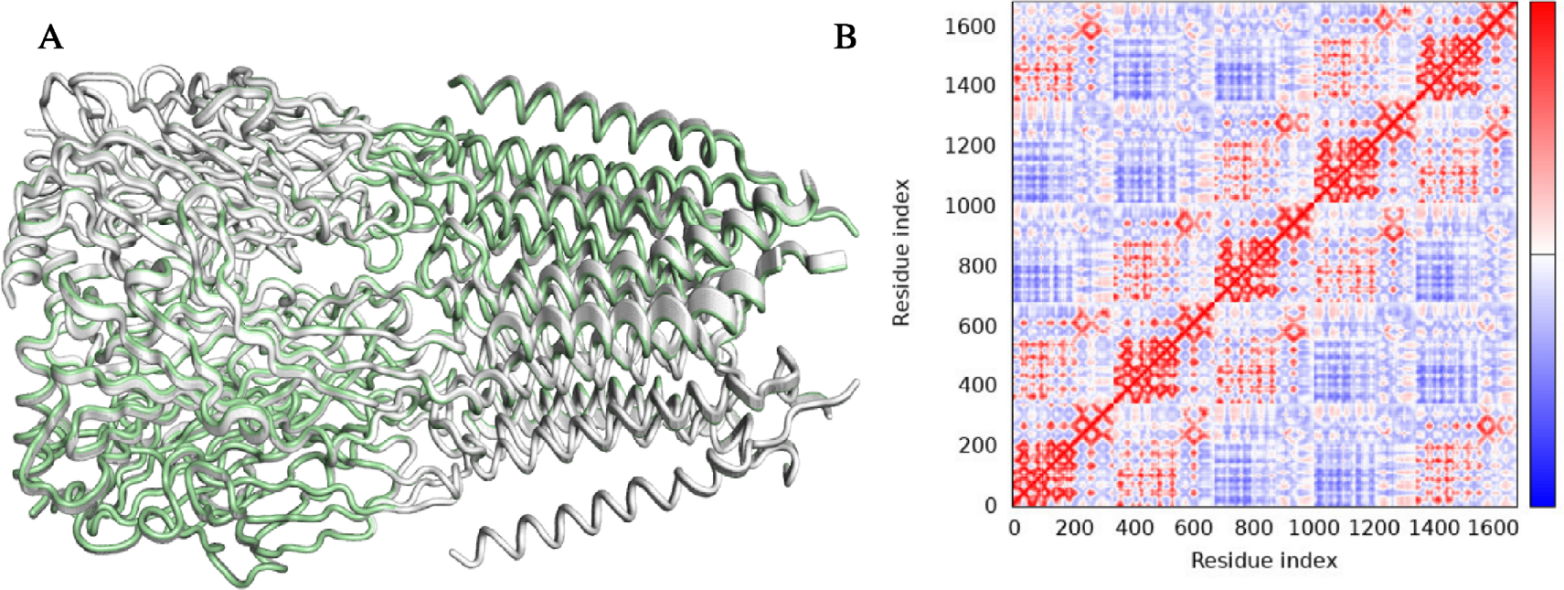
Analysis of conformational coupling and allosteric communication. (A) Structural overlap between the reference conformation (gray, PDB 6HUP) and the complex with withanolide D (green), highlighting the conformational deviations after ligand binding. (B) DCCM of alpha carbons (Cα). The red regions indicate correlated movements (same direction), while the blue regions indicate anti‐correlated movements, suggesting an allosteric communication network between the extracellular and transmembrane domains.

Global movements suggest that the peripheral helices, which determine the transmembrane domains, have the greatest mobility (yellow/red color). This region has high mobility/low deformability, indicating that this part has greater flexibility as a rigid block, but does not change its internal shape (Figure 11C) [74]. On the other hand, the opposite alpha‐helix region shows high deformability and low mobility, indicating a region that does not move large distances within the conformational space of GABAA (Figure 8D), but which undergoes significant structural distortion, indicating a possible allosteric effect of withanolide D. The observation of dynamic movements that overlap suggests the potential presence of residues within the loop and alpha‐helix regions that exhibit directional movement in a similar manner (Figure 12A). These movements are based on the graph‐based signature model integrated into the NMA model, indicating a corroboration between the results of the analysis of the protein's global flexibility [75]. In the dynamical cross‐correlation map (DCCM), it is possible to observe how the movement of residues is mechanically linked, such as residues 200 and 1400 and residues 400 and 800 (red color spectra). This finding suggests that the binding of withanolide D not only induces mobility within its binding domain but may also impact the flexibility of the protein as a whole, indicating that the extracellular domain region exhibits coordinated mobility with the transmembrane domain (Figure 12B).

## 3 Conclusions

In view of the results, the study showed that withanolide D caused anxiolytic behavior in adult zebrafish with concomitant reduction in locomotor activity, indicating partial overlap between anxiolytic‐like and sedative‐like effects depending on dose, and that the GABA_A receptor is involved in this effect, more specifically suggesting the participation of a benzodiazepine‐sensitive GABAA modulatory domain, demonstrating promising pharmacological potential. In addition, the preclinical safety of the sample was indicated, since there were no significant numbers of deaths in the 96 h of analysis. Withanolide D also prevented the impairment of memory consolidation in adult zebrafish. In silico analyses based on molecular docking estimated that withanolide D may interact with the α1γ2 domain of the GABA_A_ receptor, although it did not show interactions in common with the DZP agonist. Therefore, these results suggest a putative allosteric interaction in a functionally related region, rather than definitive occupation of the classical benzodiazepine site. Withanolide D increases the intrinsic flexibility of the GABAA receptor compared to diazepam, as shown by NMA with higher RMSF values and localized residue fluctuations associated with conformational changes. ADMET predictions showed a PAMPA profile based on moderate cell permeability, while metabolic stability affected by first‐pass biotransformation may be indicative of a pharmacological principle based on control of the daily oral dose administered. Overall, withanolide D appears to be a promising natural scaffold for the development of new anxiolytic agents, although further studies are necessary to clarify its mechanism of action and pharmacokinetic limitations.

## 4 Experimental Section

### 4.1 Vegetable Material

*A. arborescens*, belonging to the Solanaceae family was collected in August 2006 in the locality of Pico Alto, in the municipality of Guaramiranga, Ceará, Brazil, by Professor Edilberto Rocha Silveira and then cultivated at the Center for Studies and Research in Urban Agriculture—NEPAU, of the Agronomy Department of the Federal University of Ceará. The botanical material was identified by Professor Edson de Paula Nunes, and an exsiccate (number 34289) was deposited in the Prisco Bezerra Herbarium, Department of Biology, Federal University of Ceará, Brazil.

### 4.2 Obtaining the Withanolide D

Withanolide D was isolated by Batista et al. [19] from the acetone extract of *A. arborescens* leaves, and provided by Prof. Otília Deusdênia Loiola Pessoa, Coordinator of the Natural and Marine Products Laboratory of the Department of Organic and Inorganic Chemistry at the Federal University of Ceará. The ^1^H and ^13^C NMR data are from the literature [12, 16].

### 4.3 Drugs and Reagents

The drugs/reagents used in the experiments with zebrafish were diazepam (DZP; Neo Química), donepezila (DNZP, Neo Química), flumazenil (FMZ; Sandoz), dimethylsulfoxide (DMSO; dynamic) and ethanol (1% EtOH; dynamic).

### 4.4 Animal Model (Zebrafish)

Wild adult zebrafish (*Danio rerio*) were purchased from a local supplier (Fortaleza/Ceará, Brazil), with a mixture of males and females. The animals were properly acclimatized for 24 h in 10 L aquariums (*n* = 3/L), at a temperature of 25°C and pH 7.0 with dechlorinated water (ProtecPlus) and an air pump with submerged filters. The fish were monitored, as were the temperature and water quality conditions. The fish were also fed (Spirulina) ad libit ± 2°C and pH 7.0 with dechlorinated water (ProtecPlus) and an air pump with submerged filters. The fish were monitored, as were the temperature and water quality conditions. The fish were also fed (Spirulina) ad libitum 24 h before the experiments. At the end of the experiments, the animals were euthanized. The experiments were approved by the Animal Use Ethics Committee of the State University of Ceará (CEUA‐UECE; no. 04983945/2021) in accordance with the Ethical Principles for Animal Experimentation.

### 4.5 General Protocol

Zebrafish of both sexes were randomly selected for the experiments, anesthetized in ice water, transferred to a damp sponge and then treated (*n* = 6/group) with 20 µL of withanolide D at doses of 4, 20, and 40 mg/kg, diazepam (DZP; 4 mg/kg) and 3% DMSO (control group—drug diluent) or distilled water (emulsion diluent) intraperitoneally (i.p.). Next, to simulate social isolation, an anxiety‐inducing factor, the animals were placed individually in a beaker (500 mL) containing 250 mL of aquarium water and kept at rest. For intraperitoneal (i.p.) treatments, insulin syringes (0.5 mL; UltraFine BD) with a 30G needle were used.

### 4.6 Acute Toxicity 96 h

After the open field experiment, the fish (*n* = 6/group) that had been treated intraperitoneally (i.p.) with the doses evaluated (4, 20, and 40 mg/kg, 20 µL) and the negative control (3% DMSO, 20 µL) were placed individually in a beaker (500 mL) containing 250 mL of aquarium water for analysis of the mortality rate over a period of 96 h, recording the number of dead fish in each group every 24 h, with the lethal dose capable of killing 50% of the animals (LD_50_) with a 95% confidence interval according to the Organization for Economic Cooperation and Development (OECD) [65, 76].

### 4.7 Assessment of Locomotor Activity (Open Field Test—OFT)

The open field experiment was carried out with zebrafish of both sexes and they were randomly selected to assess the presence or absence of changes in the animals' motor coordination [77], whether due to anxiolytic effect, sedation, and/or muscle relaxation. Initially, the fish (*n* = 6/group) were treated intraperitoneally (i.p.) using an insulin syringe (0.5 mL; UltraFine BD) with a 30G needle to apply withanolide D (20 µL) at doses of 4, 20, and 40 mg/kg, DZP (4 mg/kg) and 3% DMSO (drug diluent). After 30 min of treatment, the animals were placed in glass Petri dishes (10 × 15 cm) containing the same water as the aquarium, marked with four quadrants, and analyzed for locomotor activity, counting the number of lines crossed (CL) by the animals during 5 min of analysis [78].

### 4.8 Anxiolytic Assessment—Light and Dark Test (LDT)

The anxiety behavior of the animals can be observed using the light/dark test, in the same way as rodents, zebrafish instinctively avoid bright areas [78, 79]. The fish were initially placed individually in a glass aquarium (30 cm × 15 cm × 20 cm) divided into two light and dark chambers to allow the animal to make an initial choice of side and then move freely between the chambers. The aquarium was filled to 3 cm with pre‐treated water free of chlorine and heavy metals, simulating a new environment, different from a conventional aquarium and with a social isolation factor that could induce anxiety behaviors [34]. The animals (*n* = 6/group) were treated intraperitoneally (20 µL) with the sample at doses of 4, 20 and 40 mg/kg each. The negative and positive control groups were composed of 3% DMSO (withanolide D diluent) and a 4 mg/kg solution of DZP, respectively. After 30 min of treatment, the animals were individually placed back in the aquarium in the clear zone, and the anxiolytic effect was measured based on the time the animals remained in the clear zone of the aquarium during 5 min of analysis [78].

### 4.9 Inhibitory Avoidance Test

In this assay, memory impairment was induced by ethanol exposure. Adult zebrafish were exposed to 1% ethanol for 20 min to promote cognitive deficits, and this group was used as the memory impairment control (EtOH group). In contrast, 3% DMSO was used exclusively as the vehicle control for withanolide D administration, since the compound was dissolved in DMSO. Therefore, DMSO 3% represents the negative control for vehicle effects, whereas 1% EtOH represents the negative control for ethanol‐induced memory impairment. As described by Blank et al. [80] and Bertoncello et al. [81], inhibitory avoidance was performed using an aquarium (28 × 14.7 × 19 cm) containing 1.3 L of dechlorinated water, divided into two equal compartments (light and dark) separated by a manually controlled guillotine door (10 × 10 cm). The dark compartment contained three metal bars (1 cm diameter), positioned vertically along the wall and spaced 3 cm apart, connected to an electrostimulation device. When activated, the device delivered an aversive stimulus (100 Hz for 5 s; 125 mA, 3 ± 0.2 V) in the dark compartment. Zebrafish (*n* = 6/group) were individually placed in 500 mL beakers for identification. Each animal was then transferred to the light compartment with the guillotine door closed and allowed to acclimate for up to 1 min. After acclimation, the door was raised, allowing access to the dark compartment. The latency to enter the dark side was recorded, and upon entry, the electrical stimulus was applied. This latency time was used as the behavioral parameter for statistical analysis. Immediately after the training session, animals were treated intraperitoneally (20 µL) with withanolide D at 4, 20, or 40 mg/kg. Additional groups received DMSO 3% (vehicle control) or donepezil (DNZP; 0.5 mg/L, i.p.) as a reference drug. Memory retention was evaluated 24 h later using the same procedure, but without electric shock. To evaluate ethanol‐induced memory impairment, the experimental design followed the protocol described by Luchiari et al. [82]. Immediately after the training session and drug administration, zebrafish were exposed by immersion to 1% ethanol for 20 min, as previously described [70, 71]. In this assay, animals (*n* = 6/group) were treated with the lowest effective dose of withanolide D (4 mg/kg), while the control groups received DMSO 3% (vehicle control) or 1% EtOH (impairment control). Memory retention was assessed 24 h after ethanol exposure, using the inhibitory avoidance test as described above, without shock.

### 4.10 Statistical Analysis

The results were expressed as mean values ± standard error of the mean for each group of 6 animals. After confirming the normality of the distribution and the homogeneity of the data, the differences between the groups were subjected to one‐way analysis of variance (one‐way ANOVA) for the open field and light/dark tests, and two‐way ANOVA for the mechanisms of action, followed by Tukey's test. All analyses were carried out using GraphPad Prism software (version 9.0.0; GraphPad Software, San Diego, CA, USA). The level of statistical significance was set at 5% (*p* < 0.05).

### 4.11 Molecular Docking Procedures

To prepare the chemical structures for the molecular docking simulations, the programs MarvinSketch, version 24.1.0, Chemaxon (https://chemaxon.com/marvin), and AutoDockTools(TM) (https://autodocksuite.scripps.edu/adt/) were used.

Initially, the two‐dimensional (2D) representation of the withanolide D and DZP chemical structures, exported in simplified molecular input line entry system (SMILES) linear notation from the PubChem repository (https://pubchem.ncbi.nlm.nih.gov/), was plotted in the MarvinSketch software. The program was configured to perform a structural optimization using the Merck molecular force field (MMFF94) method, considering the pre‐hydrogenated chemical structure, returning only the lowest energy conformation.

The chemical structure of the type‐A γ‐aminobutyric (GABA_A_) receptor, complexed to the agonist diazepam (DZP), was reported from the RCSB Protein Data Bank (https://www.rcsb.org/), deposited under PDB code ID 6HUP, classified as a membrane receptor in a *Homo sapiens* and *Escherichia Coli* expression system, whose structure was characterized by electron microscopy at a resolution of 3.58 Å [54]. In order to preserve the protein's structural integrity and maintain its biological relevance, the experimental coordinates of the protein's structure were preserved during the experimental process. To prepare the protein, the water molecules (H_2_O) and co‐crystallized DZP were removed, the hydrogens were added considering the protonation states of amino acid residues at physiological pH (approximately 7.4), and the Kollman and Gasteiger charges were computed using the AutoDockTools program [83]. Residues with basic H‐bond acceptor centers, such as histidine, were maintained in their protic state according to their protonation state at physiological pH. The dimensions of the grid‐box were set to delimit the entire conformational space of the protein, adjusted between the axes *x* = 134.39, *y* = 135.69, and *z* = 133.776 and dimensions 94 Å × 92 Å × 126 Å (*x*, *y*, and *z*). Then, the AutoDockVina code (https://vina.scripps.edu/) was configured to perform a series of 50 independent simulations of 20 poses each, for each withanolide ligand D and DPZ, ranked by affinity energy (*E*_A_) and root mean square deviation (RMSD), where the best‐pose selection criteria include E(A) ←6.0 kcal mol^−1^ and RMSD < 2.0 Å [84].

### 4.12 ADMET Predictive Study

#### 4.12.1 Molecular Lipophilicity Potential and Structure Complexity

Based on the optimized 3D structure of withanolide D, the molecular lipophilicity potential (MLP) was analyzed, as shown in Equation (1):

(1) $$\mathrm{MLP}=\sum_{i=1}^{N}F_{i}f\left(d_{ik}\right)$$

where *N* denotes the number of molecular fragments (i) distributed in the respective lipophilicity indices (*F*), while *f*(*d*) represents the function describing the spatial distance between two fragments, *i* and *k* [85]. The data obtained was correlated with the physicochemical descriptors of intrinsic lipophilicity (logP) and the topological polar surface area (TPSA) [86].

A quantitative estimate of drug‐likeness was then made using the Medicinal Chemistry Evolution, 2018 (MCE18) algorithm, as shown in Equation (2):

(2) $$\mathrm{MCE}18=\left(\mathrm{AR}+\mathrm{NAR}+\mathrm{Chiral}+\mathrm{Spiro}\frac{\mathrm{Fs}p^{3}+\mathrm{Cyc}-\mathrm{Acyc}}{1+\mathrm{Fs}p^{3}}\right)Q^{1}$$

The number of aromatic (AR) and non‐aromatic (NAR) rings, as well as the presence of chiral centers and spirocyclic (Spiro) centers, along with the distribution of the fraction of atoms with hybridization (Fsp^3^) between cyclic (Cyc) and acyclic (Acyc) substructures, were evaluated with regard to new trends in medicinal chemistry. These guidelines emphasize the prioritization of compounds with greater size and polarity, differentiating them from molecules with potential intracellular toxicity. The results were categorized according to the following thresholds: (i) MCE‐18< 45.0: low 3D complexity, characterizing old or trivial molecular skeletons. (ii) MCE‐18> 45.0: high similarity to compounds present in patents, reflecting an optimal degree of structural complexity and optimized synthetic accessibility [59].

#### 4.12.2 Cell Effective Permeability Prediction

The artificial intelligence module of the ADMET‐AI server (https://admet.ai.greenstonebio.com/) was applied to estimate the degree of passive cell permeability (*P*_app_) and clearance in human liver microsomes (ClMicro), based on the similarity test with compounds registered in the Drugbank database (https://go.drugbank.com/). For validation, properties related to ADMET were estimated based on consensus between different predictive platforms, using descriptors from the parallel artificial membrane permeability assay (PAMPA). The analyses were conducted using the ADMET‐AI (https://admet.ai.greenstonebio.com/), ADMETlab 3.0 (https://admetlab3.scbdd.com/), Deep‐PK (https://biosig.lab.uq.edu.au/deeppk/), PreADMET (https://preadmet.qsarhub.com/) and admetSAR 3.0 (https://lmmd.ecust.edu.cn/admetsar3/about.php) databases. The properties evaluated included *P*_app_ in colorectal adenocarcinoma (Caco‐2) and MDCK cells, interaction with P‐glycoprotein (P‐gp), intrinsic hepatic clearance (*Cl*_int,u_), clearance in human hepatocytes (*Cl*_Hepa_), and volume of distribution at steady state (*V*dss).

#### 4.12.3 Consensus ADMET Prediction

The qualitative ADMET profile of withanolide D was estimated using a cross‐platform consensus prediction with the following tools: ADMETlab 3.0 (https://admetlab3.scbdd.com/), ADMETboost (https://ai‐druglab.smu.edu/), ADMET‐AI (https://admet.ai.greenstonebio.com/), PreADMET (https://preadmet.webservice.bmdrc.org/), pkCSM (https://biosig.lab.uq.edu.au/pkcsm/), and admetSAR 3.0 (https://lmmd.ecust.edu.cn/admetsar3/). The results were analyzed using critical profiles of oral bioavailability, plasma protein binding (PPB), volume of distribution in steady state (Vdss), and cytochrome P450 (CYP450)‐dependent first‐pass metabolism profiles and drug‐induced liver damage.

#### 4.12.4 Site of Metabolism and Acute Rat Toxicity

Metabolic stability was predicted by identifying sites of metabolism and assessing acute toxicity in rats. To do this, the XenoSite (https://xenosite.org/) and STopTox (https://stoptox.mml.unc.edu/) servers were used to estimate the sensitivity to reactivity induced by metabolic activation, considering the dependence on CYP450 isoforms. Subsequently, the results were correlated with the lethal dose descriptors (LD_50_) in rats, considering the oral, intraperitoneal (IP), intravenous (IV) and subcutaneous (SC) routes of administration, using the GUSAR Online—Way2Drug server (https://www.way2drug.com/gusar/) [87].

#### 4.12.5 Molecular Dynamics

The best‐pose molecular docking, for withanolide D and redocked DZP, in the GABAA receptor (PDB: 6HUP), was subjected to molecular dynamics simulations based on normal mode analysis (NMA) using the iMODS server (https://imods.iqf.csic.es/), according to Equation (3):

(3) $$q_{k}^{t}=q_{k}^{0}+\sum_{k=1}^{n}a_{k}x_{k}\cos\left(2\pi \nu_{k}t+\delta_{k}\right)$$

where *a*_k_ and *δ*_k_ are the initial conditions, *x*_k_ is the *k*_o_ eigenvector, and *v*_k_ is its frequency associated with the normal mode. Normal modes are defined as a set of vectors with displacements that are perpendicular to the protein structure. High‐frequency modes indicate specific, local movement. Low‐frequency modes represent more general, group changes [88]. These changes were identified from the DCCM using the DynaMut server – BioSig Lab (https://biosig.lab.uq.edu.au/dynamut/).

## Author Contributions

**Conceptualization**: Alexandre Magno Rodrigues Teixeira, Jane Eire Silva Alencar de Menezes, Hélcio Silva dos Santos, Antonio Wlisses da Silva, Otília Deusdênia Loiola Pessoa, and Andreia Ferreira de Castro Gomes. **Experimental design and analysis**: Cléia Rocha de Sousa Feitosa, Francisco Anderson Nascimento Barros, Nicole de Abreu Bandeira, Ivana Carneiro Romão, Joilna Alves da Silva, Jéssica Bezerra Maciel, Maria Kueirislene Amâncio Ferreira, Pedro Henrique Jataí Batista, and Thais Rocha Cavalcante. **Original draft**: Cléia Rocha de Sousa Feitosa, Maria Kueirislene Amâncio Ferreira, and Jéssica Bezerra Maciel. **Software**: Emmanuel Silva Marinho, Márcia Machado Marinho, and Matheus Nunes da Rocha. **Methodology and determination of molecular structures**: Otília Deusdênia Loiola Pessoa and Pedro Henrique Jataí Batista. **Review and approval of manuscript**: all authors. **Supervision**: Alexandre Magno Rodrigues Teixeira, Jane Eire Silva Alencar de Menezes, and Hélcio Silva dos Santos.

## Funding

Otília Deusdênia Loiola Pessoa acknowledges financial support from CNPq—Universal (grant: 304817/2025‐2). Hélcio Silva dos Santos acknowledges financial support from the PQ/CNPq (grant: 306008/2022‐0) and FUNCAP‐INTERNATIONALIZATION (grant: ITR‐0214‐00060.01.00/23). Alexandre Magno Rodrigues Teixeira acknowledges financial support from the PQ/CNPq (grant: 308178/2021‐1), and from the FUNCAP (grant: UNI‐0210‐00315.01.00/23). Emmanuel Silva Marinho acknowledges financial support from FUNCAP (grant: FPD‐0213‐00369.01.00/23) and CNPQ‐PQ (grant: 309349/2025‐7).

## Ethics Statement

The bioassays were performed in accordance with the Ethical Principles of Animal Experimentation and were approved by the Ethics Committee for the Use of Animals (CEUA) of the State University of Ceará (04983945/2021).

## Conflicts of Interest

The authors declare no conflicts of interest.

## Supporting information




**Supporting File**: cbdv71284‐sup‐0001‐SuppMat.docx.

## Data Availability

The data that support the findings of this study are available from the corresponding author upon reasonable request.

## References

[1] Z. Malik , R. Parveen , B. Parveen , et al., “Anticancer Potential of Andrographolide From Andrographis paniculata (Burm.f.) Nees and Its Mechanisms of Action,” Journal of Ethnopharmacology 272 (2021): 113936, 10.1016/j.jep.2021.113936. 33610710

[2] M. M. Ahmed , H. E. Khadum , and H. M. S. Jassam , “Medicinal Herbs as Novel Therapies Against Antibiotic‐Resistant Bacteria,” Research Journal of Pharmacy and Technology 16, no. 1 (2023): 62–66, 10.52711/0974-360X.2023.00011.

[3] F. Lacouth‐Silva , C. V. Xavier , S. da S Setúbal , et al., “The Effect of 3β, 6β, 16β‐Trihydroxylup‐20(29)‐ene Lupane Compound Isolated From Combretum Leprosum Mart. On Peripheral Blood Mononuclear Cells,” BMC Complementary and Alternative Medicine 15, no. 1 (2015): 420, 10.1186/s12906-015-0948-1. 26608735 PMC4659216

[4] N. Gupta , Ramdas , N. Tiwari , et al., “Defining Accumulation of Withanamides and Withanolides in the Berries of *Withania somnifera* Linn Cultivars: UPLC‐PDA Method Development and Chemometric Analysis for Categorization for Neuroprotective Potential,” Industrial Crops and Products 225 (2025): 120501, 10.1016/j.indcrop.2025.120501.

[5] H. De‐la‐Cruz , G. Vilcapoma , and P. A. Zevallos , “Ethnobotanical Study of Medicinal Plants Used by the Andean People of Canta, Lima, Peru,” Journal of Ethnopharmacology 111, no. 2 (2007): 284–294, 10.1016/j.jep.2006.11.018. 17215096

[6] P. T. White , C. Subramanian , H. F. Motiwala , and M. S. Cohen , “Natural Withanolides in the Treatment of Chronic Diseases,” in: Anti‐inflammatory Nutraceuticals and Chronic Diseases, eds. S. C. Gupta , S. Prasad , and B. B. Aggarwal (Springer, 2016): 329–373, 10.1007/978-3-319-41334-1_14. PMC7121644 27671823

[7] E. A. Crane , W. Heydenreuter , K. R. Beck , et al., “Profiling Withanolide A for Therapeutic Targets in Neurodegenerative Diseases,” Bioorganic & Medicinal Chemistry 27, no. 12 (2019): 2508–2520, 10.1016/j.bmc.2019.03.022. 30929949

[8] S. Minguzzi , L. E. S. Barata , Y. G. Shin , et al., “Cytotoxic Withanolides From Acnistus Arborescens,” Phytochemistry 59, no. 6 (2002): 635–641, 10.1016/S0031-9422(02)00022-5. 11867095

[9] H. Kim , H.‐S. Choi , K. Han , W. Sim , H. J. Suh , and Y. Ahn , “Ashwagandha (*Withania somnifera* (L.) Dunal) Root Extract Containing Withanolide a Alleviates Depression‐Like Behavior in Mice by Enhancing the Brain‐Derived Neurotrophic Factor Pathway Under Unexpected Chronic Mild Stress,” Journal of Ethnopharmacology 340 (2025): 119224, 10.1016/j.jep.2024.119224. 39674356

[10] S. Zahiruddin , P. Basist , A. Parveen , et al., “Ashwagandha in Brain Disorders: A Review of Recent Developments,” Journal of Ethnopharmacology 257 (2020): 112876, 10.1016/j.jep.2020.112876. 32305638

[11] A. Saiyed , N. Jahan , S. F. Majeedi , and M. Roqaiya , “Medicinal Properties, Phytochemistry and Pharmacology of *Withania somnifera*: An Important Drug of Unani Medicine,” Journal of Scientific and Innovative Research 5, no. 4 (2016): 156–160, 10.31254/JSIR.2016.5412.

[12] C. P. Cordero , S. J. Morantes , A. Páez , J. Rincón , and F. A. Aristizábal , “Cytotoxicity of Withanolides Isolated From Acnistus Arborescens,” Fitoterapia 80, no. 6 (2009): 364–368, 10.1016/j.fitote.2009.05.011. 19460421

[13] H. Zhang , A. K. Samadi , M. S. Cohen , and B. N. Timmermann , “Antiproliferative Withanolides From the Solanaceae: A Structure–Activity Study,” Pure and Applied Chemistry 84, no. 6 (2012): 1353–1367, 10.1351/PAC-CON-11-10-08. PMC3789375 24098060

[14] W. Ben Bakrim , L. El Bouzidi , H. Manouze , et al., “Anti‐Amnesic Effects of withaferin A, a Steroidal Lactone Isolated From Withania adpressa, on Scopolamine‐Induced Memory Impairment in Mice,” Arabian Journal of Chemistry 15: 103529, 10.1016/j.arabjc.2021.103529.

[15] S. Maher , M. I. Choudhary , F. Saleem , et al., “Isolation of Antidiabetic Withanolides From Withania Coagulans Dunal and Their In Vitro and In Silico Validation,” Biology 9, no. 8 (2020): 197, 10.3390/biology9080197. 32751610 PMC7464911

[16] V. Roumy , M. Biabiany , T. Hennebelle , et al., “Antifungal and Cytotoxic Activity of Withanolides From Acnistus Arborescens,” Journal of Natural Products 73, no. 7 (2010): 1313–1317, 10.1021/np100201p. 20590148

[17] A. I. V. Maia , R. Braz‐Filho , E. R. Silveira , C. A. de Simone , and O. D. L. Pessoa , “Further Withaphysalin Derivatives From *Acnistus arborescens* ,” Helvetica Chimica Acta 95, no. 8 (2012): 1387–1394, 10.1002/hlca.201100386.

[18] M. L. Veras , M. Z. B. Bezerra , R. Braz‐Filho , et al., “Cytotoxic Epimeric Withaphysalins From Leaves of *Acnistus arborescens* ,” Planta Medica 70, no. 06 (2004): 551–555, 10.1055/s-2004-827156. 15241891

[19] P. H. J. Batista , K. S. B. de Lima , F. C. L. Pinto , et al., “Withanolides From Leaves of Cultivated *Acnistus arborescens* ,” Phytochemistry 130 (2016) 321–327, 10.1016/j.phytochem.2016.07.003. 27498045

[20] M. W. Baig , B. Nasir , D. Waseem , M. Majid , M. Z. I. Khan , and I.‐U. Haq , “Withametelin: A Biologically Active Withanolide in Cancer, Inflammation, Pain and Depression,” Saudi Pharmaceutical Journal 28, no. 12 (2020): 1526–1537, 10.1016/j.jsps.2020.09.021. 33424246 PMC7783102

[21] C. K. Benneh , R. P. Biney , P. K. Mante , A. Tandoh , D. W. Adongo , and E. Woode , “Maerua Angolensis Stem Bark Extract Reverses Anxiety and Related Behaviours in Zebrafish—Involvement of GABAergic and 5‐HT Systems,” Journal of Ethnopharmacology 207 (2017): 129–145, 10.1016/j.jep.2017.06.012. 28645783

[22] C. Salzman , “Do Benzodiazepines Cause Alzheimer's Disease?,” American Journal of Psychiatry 177, no. 6 (2020): 476–478, 10.1176/appi.ajp.2020.20040375. 32475136

[23] S. D. Hood , A. Norman , D. A. Hince , J. K. Melichar , and G. K. Hulse , “Benzodiazepine Dependence and Its Treatment With Low Dose Flumazenil,” British Journal of Clinical Pharmacology 77, no. 2 (2014): 285–294, 10.1111/bcp.12023. 23126253 PMC4014019

[24] M. Majeed , K. Nagabhushanam , and L. Mundkur , “A Standardized Ashwagandha Root Extract Alleviates Stress, Anxiety, and Improves Quality of Life in Healthy Adults by Modulating Stress Hormones: Results From a Randomized, Double‐Blind, Placebo‐Controlled Study,” Medicine 102, no. 41 (2023): e35521, 10.1097/md.0000000000035521. 37832082 PMC10578737

[25] J. A. Soria Lopez , H. M. González , and G. C. Léger , Chapter 13‐Alzheimer's Disease, in: Handbook of Clinical Neurology, eds. S.T. Dekosky and S. Asthana 167 (Elsevier, 2019): 231–255, 10.1016/B978-0-12-804766-8.00013-3. 31753135

[26] A. K. Shahid , B. K. Sher , S. Zarbad , and M. A. Abdullah , “Withanolides: Biologically Active Constituents in the Treatment of Alzheimer's Disease,” Medicinal Chemistry 12, no. 3 (2016): 238–256, 10.2174/1573406411666151030112314. 26527154

[27] A. Bashir , M. Nabi , N. Tabassum , S. Afzal , and M. Ayoub , “An Updated Review on Phytochemistry and Molecular Targets of *Withania somnifera* (L.) Dunal (Ashwagandha),” Frontiers in Pharmacology 14 (2023): 2023, 10.3389/fphar.2023.1049334. PMC10090468 37063285

[28] A. G. Atanasov , B. Waltenberger , E.‐M. Pferschy‐Wenzig , et al., “Discovery and Resupply of Pharmacologically Active Plant‐Derived Natural Products: A Review,” Biotechnology Advances 33, no. 8 (2015): 1582–1614, 10.1016/j.biotechadv.2015.08.001. 26281720 PMC4748402

[29] F. E. A. Magalhães , C. Á. P. B. de Sousa , S. A. A. R. Santos , et al., “Adult Zebrafish (Danio rerio): An Alternative Behavioral Model of Formalin‐Induced Nociception,” Zebrafish 14, no. 5 (2017): 422–429, 10.1089/zeb.2017.1436. 28704145

[30] K. Mhalhel , M. Sicari , L. Pansera , et al., “Zebrafish: A Model Deciphering the Impact of Flavonoids on Neurodegenerative Disorders,” Cells 12, no. 2 (2023): 252, 10.3390/cells12020252. 36672187 PMC9856690

[31] N. Assad , W. L. Luz , M. Santos‐Silva , et al., “Acute Restraint Stress Evokes Anxiety‐Like Behavior Mediated by Telencephalic Inactivation and GabAergic Dysfunction in Zebrafish Brains,” Scientific Reports 10, no. 1 (2020): 5551, 10.1038/s41598-020-62077-w. 32218457 PMC7099036

[32] J. B. Maciel , H. R. Liberato , and A. W. da Silva , “Withanicandrin Isolated From Datura Ferox Promotes Antinociception by Modulating the Asics and TRPS Channels and Anti‐Inflammation in Adult Zebrafish,” Chemistry & Biodiversity 21, no. 7 (2024): e202400538, 10.1002/cbdv.202400538. 38639566

[33] T. K. Mildenberger , M. H. Taylor , and M. Wolff , “TropFishR: An R Package for Fisheries Analysis With Length‐Frequency Data,” Methods in Ecology and Evolution 8, no. 11 (2017): 1520–1527, 10.1111/2041-210X.12791.

[34] D. L. Gebauer , N. Pagnussat , Â. L. Piato , I. C. Schaefer , C. D. Bonan , and D. R. Lara , “Effects of Anxiolytics in Zebrafish: Similarities and Differences Between Benzodiazepines, Buspirone and Ethanol,” Pharmacology Biochemistry and Behavior 99, no. 3 (2011): 480–486, 10.1016/j.pbb.2011.04.021. 21570997

[35] A. Kumar and H. Kalonia , “Protective Effect of *Withania somnifera* Dunal on the Behavioral and Biochemical Alterations in Sleep‐Disturbed Mice (Grid Over water suspended method),” Indian Journal of Experimental Biology 45, no. 6 (2007): 524–528. 17585686

[36] M. Candelario , E. Cuellar , J. M. Reyes‐Ruiz , et al., “Direct Evidence for GABAergic Activity of *Withania somnifera* on Mammalian Ionotropic GABAA and GABAρ Receptors,” Journal of Ethnopharmacology 171 (2015): 264–272, 10.1016/j.jep.2015.05.058. 26068424

[37] B. S. Alex , A. C. Kadine , S. Amala , and M. W. Kirsten , “Effects of *Withania somnifera*(Ashwagandha) on Stress and the Stress‐Related Neuropsychiatric Disorders Anxiety, Depression, and Insomnia,” Current Neuropharmacology 19, no. 9 (2021): 1468–1495, 10.2174/1570159X19666210712151556. 34254920 PMC8762185

[38] M. I. Choudhary , S. Yousuf , S. A. Nawaz , S. Ahmed , and R. Atta ur , “Cholinesterase Inhibiting Withanolides From *Withania somnifera* ,” Chemical and Pharmaceutical Bulletin 52, no. 11 (2004): 1358–1361, 10.1248/cpb.52.1358. 15520512

[39] S. P. Patil , S. Maki , S. A. Khedkar , A. C. Rigby , and C. Chan , “Withanolide A and Asiatic Acid Modulate Multiple Targets Associated With Amyloid‐β Precursor Protein Processing and Amyloid‐β Protein Clearance,” Journal of Natural Products 73, no. 7 (2010) 1196–1202, 10.1021/np900633j. 20553006 PMC2917495

[40] E. A. Eckman and C. B. Eckman , “Aβ‐Degrading Enzymes: Modulators of Alzheimer's Disease Pathogenesis and Targets for Therapeutic Intervention,” Biochemical Society Transactions 33, no. 5 (2005): 1101–1105, 10.1042/bst0331101. 16246055

[41] V. B. Dorfman , L. Pasquini , M. Riudavets , et al., “Differential Cerebral Deposition of IDE and NEP in Sporadic and Familial Alzheimer's Disease,” Neurobiology of Aging 31, no. 10 (2010): 1743–1757, 10.1016/j.neurobiolaging.2008.09.016. 19019493 PMC3266723

[42] E. K. Perry , B. E. Tomlinson , G. Blessed , K. Bergmann , P. H. Gibson , and R. H. Perry , “Correlation of Cholinergic Abnormalities With Senile Plaques and Mental Test Scores in Senile Dementia,” BMJ 2, no. 6150 (1978): 1457–1459, 10.1136/bmj.2.6150.1457. 719462 PMC1608703

[43] H. Hampel , M.‐M. Mesulam , A. C. Cuello , et al., “The Cholinergic System in the Pathophysiology and Treatment of Alzheimer's Disease,” Brain 141, no. 7 (2018): 1917–1933, 10.1093/brain/awy132. 29850777 PMC6022632

[44] B. J. Cummings and C. W. Cotman , “Image Analysis of β‐amyloid Load in Alzheimer's Disease and Relation to Dementia Severity,” Lancet 346 (8989): 1524–1528, 10.1016/S0140-6736(95)92053-6. 7491048

[45] W. Q. Mota , I. S. Lobão , J. V. D. Cunha , et al., “Aspectos Clínicos e Moleculares do Alzheimer—Revisão de Literatura,” Brazilian Journal of Health Review 7, no. 2 (2024): 1–20, 10.34119/bjhrv7n2-465.

[46] R. Schliebs , A. Liebmann , S. K. Bhattacharya , A. Kumar , S. Ghosal , and V. Bigl , “Systemic Administration of Defined Extracts From *Withania somnifera* (Indian ginseng) and Shilajit Differentially Affects Cholinergic but Not Glutamatergic and GABAergic Markers in Rat Brain,” Neurochemistry International 30, no. 2 (1997): 181–190, 10.1016/S0197-0186(96)00025-3. 9017665

[47] M. H. Veerendra Kumar and Y. K. Gupta , “Effect of Different Extracts of *Centella asiatica* on Cognition and Markers of Oxidative Stress in Rats,” Journal of Ethnopharmacology 79, no. 2 (2002): 253–260, 10.1016/S0378-8741(01)00394-4. 11801389

[48] E. Tamagno , P. Bardini , A. Obbili , et al., “Oxidative Stress Increases Expression and Activity of BACE in NT2 Neurons,” Neurobiology of Disease 10, no. 3 (2002): 279–288, 10.1006/nbdi.2002.0515. 12270690

[49] S. Patil , L. Sheng , A. Masserang , and C. Chan , “Palmitic Acid‐Treated Astrocytes Induce BACE1 Upregulation and Accumulation of C‐Terminal Fragment of APP in Primary Cortical Neurons,” Neuroscience Letters 406, no. 1 (2006): 55–59, 10.1016/j.neulet.2006.07.015. 16904262

[50] F. R. S. Mendes , A. W. da Silva , M. K. A. Ferreira , et al., “GABA _A_ and Serotonergic Receptors Participation in Anxiolytic Effect of Chalcones in Adult Zebrafish,” Journal of Biomolecular Structure and Dynamics 41 (2023): 12426–12444, 10.1080/07391102.2023.2167116. 36644862

[51] Y. Cao , H. Yan , G. Yu , and R. Su , “Flumazenil‐Insensitive Benzodiazepine Binding Sites in GABAA Receptors Contribute to Benzodiazepine‐Induced Immobility in Zebrafish Larvae,” Life Sciences 239 (2019): 117033, 10.1016/j.lfs.2019.117033. 31697950

[52] D. Yusuf , A. M. Davis , G. J. Kleywegt , and S. Schmitt , “An Alternative Method for the Evaluation of Docking Performance: RSR vs RMSD,” Journal of Chemical Information and Modeling 48, no. 7 (2008): 1411–1422, 10.1021/ci800084x. 18598022

[53] S. Shityakov and C. Forster , “In Silico Predictive Model to Determine Vector‐Mediated Transport Properties for the Blood–Brain Barrier Choline Transporter,” Advances and Applications in Bioinformatics and Chemistry 7 (2014): 23–36, 10.2147/aabc.S63749. 25214795 PMC4159400

[54] S. Masiulis , R. Desai , T. Uchański , et al., “GABAA Receptor Signalling Mechanisms Revealed by Structural Pharmacology,” Nature 565, no. 7740 (2019): 454–459, 10.1038/s41586-018-0832-5. 30602790 PMC6370056

[55] A. Imberty , K. D. Hardman , J. P. Carver , and S. Perez , “Molecular Modelling of Protein‐Carbohydrate Interactions. Docking of Monosaccharides in the Binding Site of Concanavalin A,” Glycobiology 1, no. 6 (1991): 631–642, 10.1093/glycob/1.6.631. 1822243

[56] E. Persch , O. Dumele , and F. Diederich , “Molecular Recognition in Chemical and Biological Systems,” Angewandte Chemie International Edition 54, no. 11 (2015): 3290–3327, 10.1002/anie.201408487. 25630692

[57] P. Pandey , A. Zagzoog , R. B. Laprairie , W. M. Neal , R. J. Doerksen , and A. G. Chittiboyina , “Determination of the Negative Allosteric Binding Site of Cannabidiol at the CB1 Receptor: A Combined Computational and Site‐Directed Mutagenesis Study,” ACS Chemical Neuroscience 16 (2025): 311–328, 10.1021/acschemneuro.4c00343. 39812521

[58] J. B. GC , C. T. Szlenk , A. Diyaolu , et al., “Allosteric Modulation of α1β3γ2 GABAA Receptors by Farnesol Through the Neurosteroid Sites,” Biophysical Journal 122, no. 5 (2023): 849–867, 10.1016/j.bpj.2023.01.032. 36721367 PMC10027449

[59] Y. A. Ivanenkov , B. A. Zagribelnyy , and V. A. Aladinskiy , “Are We Opening the Door to a New Era of Medicinal Chemistry or Being Collapsed to a Chemical Singularity?,” Journal of Medicinal Chemistry 62, no. 22 (2019): 10026–10043, 10.1021/acs.jmedchem.9b00004. 31188596

[60] J. D. Hughes , J. Blagg , D. A. Price , et al., “Physiochemical Drug Properties Associated With In Vivo Toxicological Outcomes,” Bioorganic & Medicinal Chemistry Letters 18, no. 17 (2008): 4872–4875, 10.1016/j.bmcl.2008.07.071. 18691886

[61] T. T. Wager , X. Hou , P. R. Verhoest , and A. Villalobos , “Central Nervous System Multiparameter Optimization Desirability: Application in Drug Discovery,” ACS Chemical Neuroscience 7, no. 6 (2016): 767–775, 10.1021/acschemneuro.6b00029. 26991242

[62] M. Pettersson , X. Hou , M. Kuhn , T. T. Wager , G. W. Kauffman , and P. R. Verhoest , “Quantitative Assessment of the Impact of Fluorine Substitution on P‐Glycoprotein (P‐gp) Mediated Efflux, Permeability, Lipophilicity, and Metabolic Stability,” Journal of Medicinal Chemistry 59, no. 11 (2016): 5284–5296, 10.1021/acs.jmedchem.6b00027. 27228214

[63] K. Swanson , P. Walther , J. Leitz , et al., “ADMET‐AI: A Machine Learning ADMET Platform for Evaluation of Large‐Scale Chemical Libraries,” Bioinformatics 40, no. 7 (2024): btae416, 10.1093/bioinformatics/btae416. 38913862 PMC11226862

[64] E. V. Radchenko , A. S. Dyabina , V. A. Palyulin , and N. S. Zefirov , “Prediction of Human Intestinal Absorption of Drug Compounds,” Russian Chemical Bulletin 65, no. 2 (2016): 576–580, 10.1007/s11172-016-1340-0.

[65] T. W. Johnson , K. R. Dress , and M. Edwards , “Using the Golden Triangle to Optimize Clearance and Oral Absorption,” Bioorganic & Medicinal Chemistry Letters 19, no. 19 (2009): 5560–5564, 10.1016/j.bmcl.2009.08.045. 19720530

[66] N.‐N. Wang , J. Dong , Y.‐H. Deng , et al., “ADME Properties Evaluation in Drug Discovery: Prediction of Caco‐2 Cell Permeability Using a Combination of NSGA‐II and Boosting,” Journal of Chemical Information and Modeling 56 (2016): 763–773, 10.1021/acs.jcim.5b00642. 27018227

[67] A. K. Mandagere , T. N. Thompson , and K.‐K. Hwang , “Graphical Model for Estimating Oral Bioavailability of Drugs in Humans and Other Species From Their Caco‐2 Permeability and In Vitro Liver Enzyme Metabolic Stability Rates,” Journal of Medicinal Chemistry 45, no. 2 (2002): 304–311, 10.1021/jm010152k. 11784135

[68] A. Hersey , “ChEMBL Deposited Data Set—AZ_dataset”, EMBL‐EBI, fev, (2015), 10.6019/chembl3301361.

[69] D. E. V. Pires , L. M. Kaminskas , and D. B. Ascher , “Prediction and Optimization of Pharmacokinetic and Toxicity Properties of the Ligand”, in Computational Drug Discovery and Design, eds. M. Gore and U. B. Jagtap 1762 (Humana Press, 2018): 271–284, 10.1007/978-1-4939-7756-7_14. 29594777

[70] X. Ma , C. Chen , and J. Yang , “Predictive Model of Blood‐Brain Barrier Penetration of Organic Compounds1,” Acta Pharmacologica Sinica 26 (2005): 500–512, 10.1111/j.1745-7254.2005.00068.x. 15780201

[71] K. Yu , X. Geng , M. Chen , et al., “High Daily Dose and Being a Substrate of Cytochrome P450 Enzymes Are Two Important Predictors of Drug‐Induced Liver Injury,” Drug Metabolism and Disposition 42 (2014): 744–750, 10.1124/dmd.113.056267. 24464804

[72] M. Zheng , X. Luo , Q. Shen , et al., “Site of Metabolism Prediction for Six Biotransformations Mediated by Cytochromes P450,” Bioinformatics 25, no. 10 (2009): 1251–1258, 10.1093/bioinformatics/btp140. 19286831

[73] T. B. Hughes , G. P. Miller , and S. J. Swamidass , “Modeling Epoxidation of Drug‐Like Molecules With a Deep Machine Learning Network,” ACS Central Science 1, no. 4 (2015): 168–180, 10.1021/acscentsci.5b00131. 27162970 PMC4827534

[74] J. A. Kovacs , P. Chacón , and R. Abagyan , “Predictions of Protein Flexibility: First‐Order Measures,” Proteins: Structure, Function, and Bioinformatics 56 (2004): 661–668, 10.1002/prot.20151. 15281119

[75] C. H. Rodrigues , D. E. Pires , and D. B. Ascher , “DynaMut: Predicting the Impact of Mutations on Protein Conformation, Flexibility and Stability,” Nucleic Acids Research 46 (2018): W350–W355, 10.1093/nar/gky300. 29718330 PMC6031064

[76] OECD, F., Acute Toxicity Test, Effects on Biotic Systems, OECD Guideline for testing of chemicals, Section 2 (2019): 1–9.

[77] M. K. A. Ferreira , A. W. da Silva , A. L. dos Santos Moura , et al., “Chalcones Reverse the Anxiety and Convulsive Behavior of Adult Zebrafish,” Epilepsy & Behavior 117 (2021): 107881, 10.1016/j.yebeh.2021.107881. 33711684

[78] N. G. G. Gonçalves , J. I. F. de Araújo , F. E. A. Magalhães , et al., “Protein Fraction From Artocarpus Altilis Pulp Exhibits Antioxidant Properties and Reverses Anxiety Behavior in Adult Zebrafish via the Serotoninergic System,” Journal of Functional Foods 66 (2020): 103772, 10.1016/j.jff.2019.103772.

[79] R. E. Blaser , L. Chadwick , and G. C. McGinnis , “Behavioral Measures of Anxiety in Zebrafish (Danio rerio),” Behavioural Brain Research 208, no. 1 (2010): 56–62, 10.1016/j.bbr.2009.11.009. 19896505

[80] M. Blank , L. D. Guerim , R. F. Cordeiro , and M. R. M. Vianna , “A One‐Trial Inhibitory Avoidance Task to Zebrafish: Rapid Acquisition of an NMDA‐Dependent Long‐Term Memory,” Neurobiology of Learning and Memory 92, no. 4 (2009): 529–534, 10.1016/j.nlm.2009.07.001. 19591953

[81] K. T. Bertoncello , T. E. Müller , B. D. Fontana , F. Franscescon , G. L. B. Filho , and D. B. Rosemberg , “Taurine Prevents Memory Consolidation Deficits in a Novel Alcohol‐Induced Blackout Model in Zebrafish,” Progress in Neuro‐Psychopharmacology and Biological Psychiatry 93 (2019): 39–45, 10.1016/j.pnpbp.2019.03.006. 30880191

[82] A. C. Luchiari , D. C. Salajan , and R. Gerlai , “Acute and Chronic Alcohol Administration: Effects on Performance of Zebrafish in a Latent Learning Task,” Behavioural Brain Research 282 (2015): 76–83, 10.1016/j.bbr.2014.12.013. 25557800 PMC4339105

[83] V. M. de Oliveira , M. N. da Rocha , C. H. A. Roberto , et al., “Insights of Structure‐Based Virtual Screening and MPO‐Based SAR Analysis of Berberine‐Benzimidazole Derivatives Against Parkinson Disease,” Journal of Molecular Structure 1302 (2024): 137453, 10.1016/j.molstruc.2023.137453.

[84] E. M. Marinho , J. Batista de Andrade Neto , J. Silva , et al., “Virtual Screening Based on Molecular Docking of Possible Inhibitors of Covid‐19 Main Protease,” Microbial Pathogenesis 148 (2020): 104365, 10.1016/j.micpath.2020.104365. 32619669 PMC7834391

[85] N. Oberhauser , A. Nurisso , and P.‐A. Carrupt , “MLP Tools: A PyMOL Plugin for Using the Molecular Lipophilicity Potential in Computer‐Aided Drug Design,” Journal of Computer‐Aided Molecular Design 28, no. 5 (2014): 587–596, 10.1007/s10822-014-9744-0. 24777339

[86] M. Nunes da Rocha , M. Machado Marinho , H. Silva dos Santos , et al., “Structure‐Based Virtual Screening of New Antitumor Natural Berberines: Bioactivity Against Pancreas Cancer by HIF1 Inhibition Effect,” Journal of Molecular Structure 1294 (2023): 136508, 10.1016/j.molstruc.2023.136508.

[87] J. P. O. Lima , A. M. da Fonseca , G. S. Marinho , et al., “De Novo Design of Bioactive Phenol and Chromone Derivatives for Inhibitors of Spike Glycoprotein of SARS‐CoV‐2 in Silico,” 3 Biotech 13, no. 9 (2023): 301, 10.1007/s13205-023-03695-9. PMC10425314 37588795

[88] J. R. López‐Blanco , J. I. Aliaga , E. S. Quintana‐Ortí , and P. Chacón , “iMODS: Internal Coordinates Normal Mode Analysis Server,” Nucleic Acids Research 42 (2014): W271–W276, 10.1093/nar/gku339. 24771341 PMC4086069

