# Supplementary material for: Anxiolytic and Memory Protective Effects of Withanolide D Isolated From Acnistus arborescens in Adult Zebrafish
Source: Chem Biodivers. 2026 Apr 30;23:e02272. doi: 10.1002/cbdv.202502272 (PMC13130859; doi:10.1002/cbdv.202502272)
Supplement: Supplementary file 1 — Supporting File: cbdv71284‐sup‐0001‐SuppMat.docx. [file CBDV-23-e02272-s001.docx]

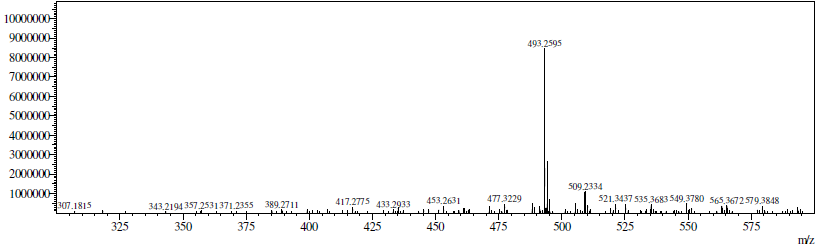


Figure S1. Mass spectrum of Withanolide D


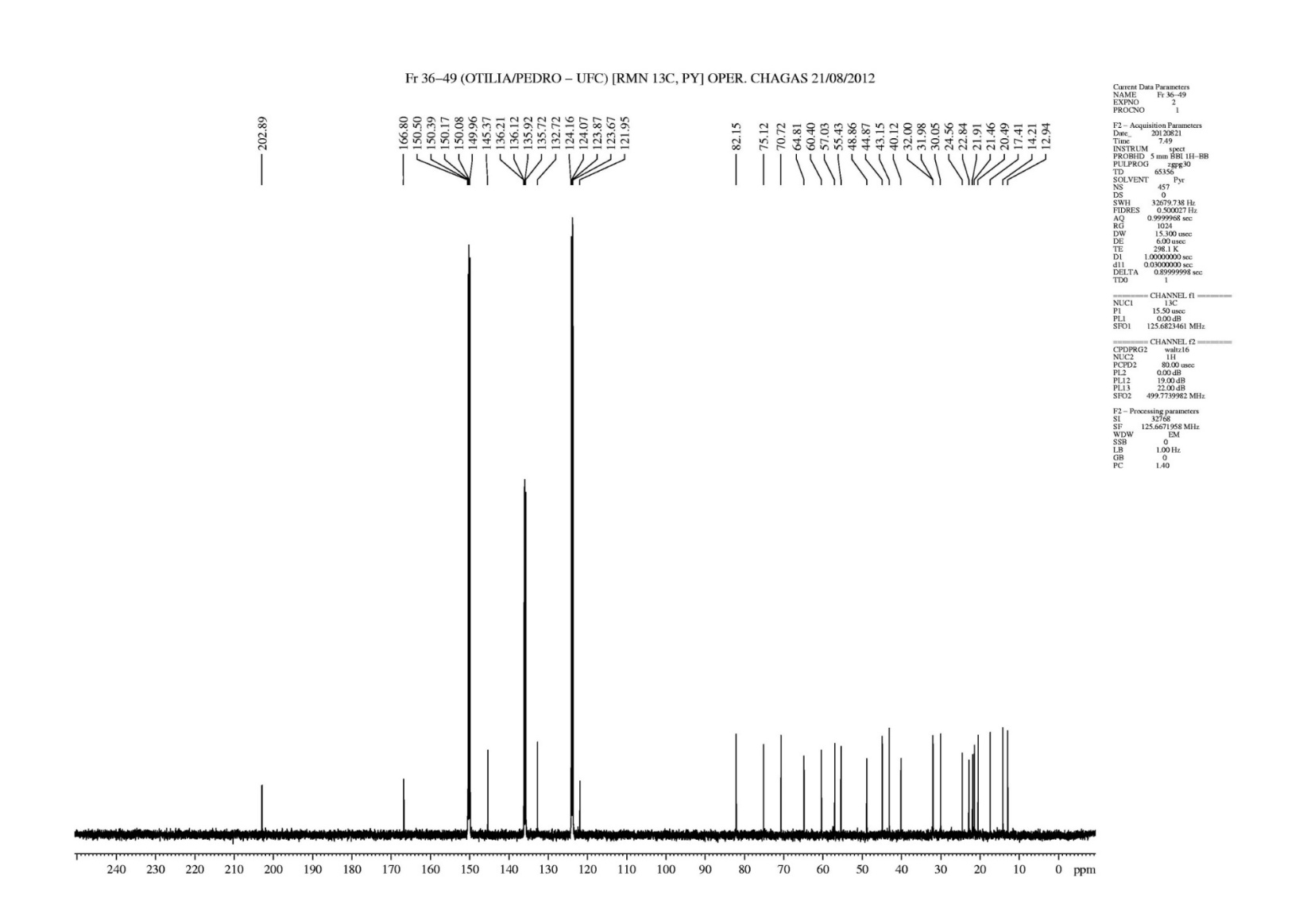


Figure S2. Carbon 13 spectrum of Withanolide D


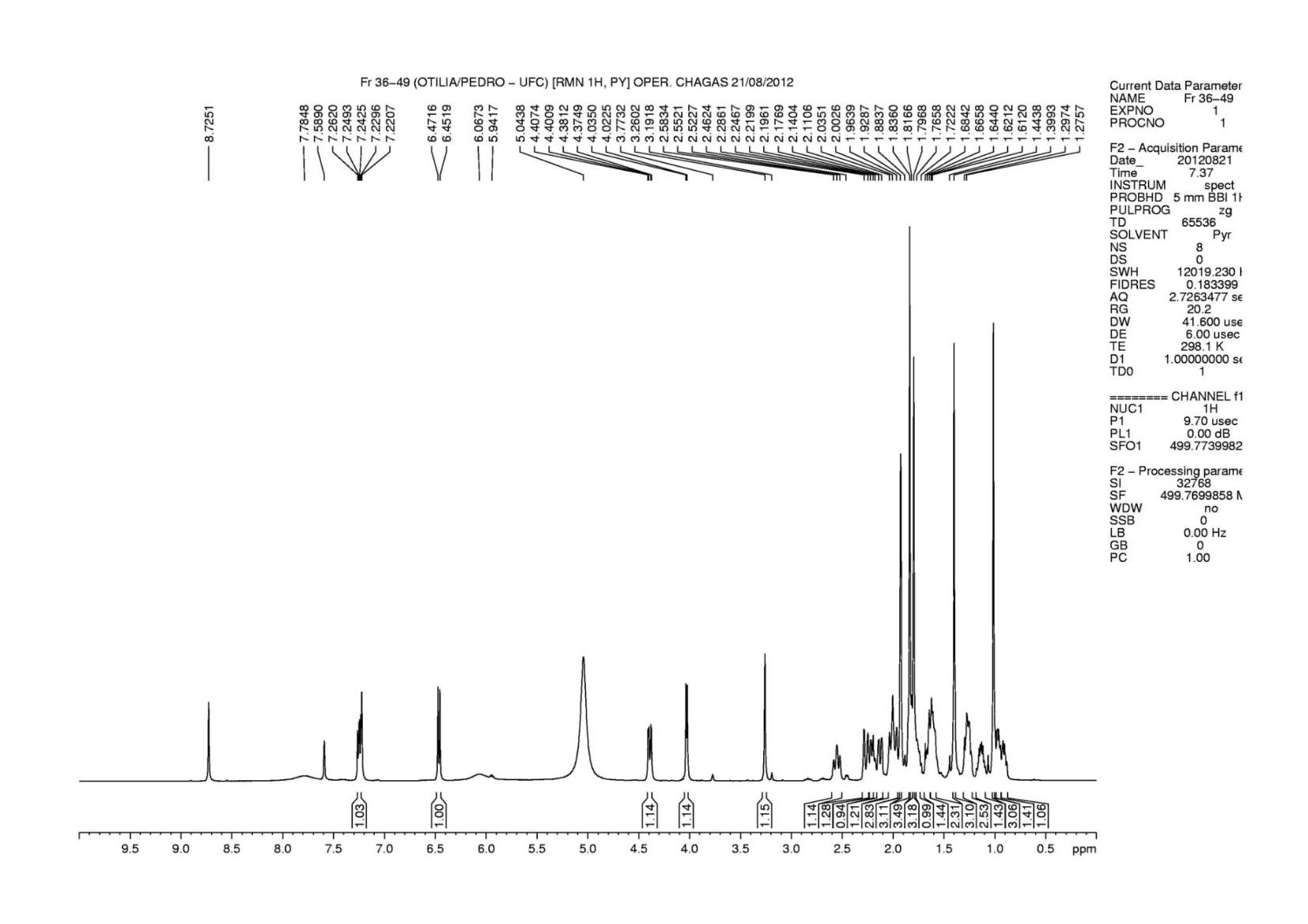


Figure S3. ^1^H NMR spectrum of Withanolide D
